# Diverse Inhibitor Chemotypes Targeting *Trypanosoma cruzi* CYP51

**DOI:** 10.1371/journal.pntd.0001736

**Published:** 2012-07-31

**Authors:** Shamila S. Gunatilleke, Claudia M. Calvet, Jonathan B. Johnston, Chiung-Kuang Chen, Grigori Erenburg, Jiri Gut, Juan C. Engel, Kenny K. H. Ang, Joseph Mulvaney, Steven Chen, Michelle R. Arkin, James H. McKerrow, Larissa M. Podust

**Affiliations:** 1 Sandler Center for Drug Discovery, University of California San Francisco, San Francisco, California, United States of America; 2 Department of Pathology, University of California San Francisco, San Francisco, California, United States of America; 3 Cellular Ultra-Structure Laboratory, Oswaldo Cruz Institute (IOC), FIOCRUZ, Rio de Janeiro, Rio de Janeiro, Brazil; 4 Department of Pharmaceutical Chemistry, University of California San Francisco, San Francisco, California, United States of America; 5 King's University College at the University of Western Ontario, London, Ontario, Canada; 6 Department of Medicine, University of California San Francisco, San Francisco, California, United States of America; 7 Small Molecule Discovery Center, University of California San Francisco, San Francisco, California, United States of America; Northeastern University, United States of America

## Abstract

**Background:**

Chagas Disease, a WHO- and NIH-designated neglected tropical disease, is endemic in Latin America and an emerging infection in North America and Europe as a result of population moves. Although a major cause of morbidity and mortality due to heart failure, as well as inflicting a heavy economic burden in affected regions, Chagas Disease elicits scant notice from the pharmaceutical industry because of adverse economic incentives. The discovery and development of new routes to chemotherapy for Chagas Disease is a clear priority.

**Methodology/Principal Findings:**

The similarity between the membrane sterol requirements of pathogenic fungi and those of the parasitic protozoon *Trypanosoma cruzi*, the causative agent of Chagas human cardiopathy, has led to repurposing anti-fungal azole inhibitors of sterol 14α-demethylase (CYP51) for the treatment of Chagas Disease. To diversify the therapeutic pipeline of anti-Chagasic drug candidates we exploited an approach that included directly probing the *T. cruzi* CYP51 active site with a library of synthetic small molecules. Target-based high-throughput screening reduced the library of ∼104,000 small molecules to 185 hits with estimated nanomolar K*_D_* values, while cross-validation against *T. cruzi*-infected skeletal myoblast cells yielded 57 active hits with EC_50_ <10 µM. Two pools of hits partially overlapped. The top hit inhibited *T. cruzi* with EC_50_ of 17 nM and was trypanocidal at 40 nM.

**Conclusions/Significance:**

The hits are structurally diverse, demonstrating that CYP51 is a rather permissive enzyme target for small molecules. Cheminformatic analysis of the hits suggests that CYP51 pharmacology is similar to that of other cytochromes P450 therapeutic targets, including thromboxane synthase (CYP5), fatty acid ω-hydroxylases (CYP4), 17α-hydroxylase/17,20-lyase (CYP17) and aromatase (CYP19). Surprisingly, strong similarity is suggested to glutaminyl-peptide cyclotransferase, which is unrelated to CYP51 by sequence or structure. Lead compounds developed by pharmaceutical companies against these targets could also be explored for efficacy against *T. cruzi*.

## Author Summary

Chagas Disease is the leading cause of heart disease in Latin America and an emerging infection in Europe and North America. The clinical presentation of Chagas Disease arises from infection by the protozoan parasite *Trypanosoma cruzi*, which leads to progressive cardiomyopathy. No vaccine is available and chemotherapeutic options are limited to the drugs benznidazole and nifurtimox, which are used during the acute phase but may cause severe gastrointestinal and neurological side effects and are not commonly used in the chronic phase. Neither drug is approved by the FDA for use in the United States. The need for effective new therapy is urgent. A validated therapeutic target in *T. cruzi* is CYP51, an essential enzyme in the sterol biosynthesis pathway. We report results of high-throughput screening of small molecules directly against CYP51, confirmed by *in vitro* medium-throughput screening of the hits against *T. cruzi*-infected mammalian cells. We have identified a potent T. cruzi inhibitor as well as a diverse collection of low molecular weight hits with high affinity to CYP51. We have applied computational chemistry to relate CYP51 to other pharmacologic targets. This analysis allowed us to identify molecules already produced by pharmaceutical companies for future experimental testing against *T. cruzi*.

## Introduction

The parasitic protozoon *Trypanosoma cruzi*, the etiological agent of Chagas Disease, is naturally transmitted by blood-sucking bugs of the subfamily *Triatominae* [1]. The range of clinically approved drugs for both the acute and chronic stage of the disease is limited and new medications are urgently needed [2]–[4]. The integrity of the parasite's cell membrane depends on specific endogenously synthesized 24-methylated sterols [5], membrane building blocks which cannot be entirely substituted by cholesterol scavenged from the host in the clinically relevant amastigote stage. The sterol biosynthesis pathway in *T. cruzi* is thus essential for the survival of the replicative intracellular amastigote stage in infected human hosts [5]–[7]. The similarity between membrane sterol requirements of *T. cruzi* and those of pathogenic fungi has led to the idea of repurposing anti-fungal azole and triazole drugs for Chagas Disease therapy. For example, posaconazole and ravuconazole target sterol 14α-demethylase (CYP51), the key enzyme in sterol biosynthesis, and therefore have potential as therapeutics [8]–[12]. Inhibitors of CYP51 deplete endogenous 24-methylated sterols in the intracellular amastigote stage, resulting in blebbing of the cell membrane, deterioration of internal membranes and finally in *T. cruzi* cell death [13], [14].

Commercial anti-fungal drugs of the “conazole” pedigree are derived from miconazole that was mainly used for topical treatment of fungal infections [15]. Posaconazole, the most recent member of this family to be marketed, is considered not only a valuable addition to the therapeutic armamentarium against systemic life-threatening fungal infections [16], [17], but also is a clinical trial candidate for Chagas Disease. The anti-chagasic potency of posaconazole has been demonstrated in an animal model of *T. cruzi* infection [9], [18], [19] and in humans [12]. However, potential issues of drug resistance, cost and safety mar the prospective utility of azole drugs. To broaden the range of CYP51 inhibitors that could be used most efficaciously to treat patients with Chagas Disease, we employed an approach that relies on directly probing the CYP51 active site with diverse compounds to define the chemical and structural space accessible to inhibitors. This approach is based on advances in high throughput screening, medicinal chemistry, structural biology, and cheminformatics, and with it we have identified a broad selection of promising molecular scaffolds.

As a member of the P450 family, CYP51 shares the common spectral property of P450 enzymes in which the ferric Fe Soret band is shifted following the displacement (type I shift) or replacement (type II shift) of a water molecule, a weak axial ligand, by a stronger one, usually one containing a nitrogen aliphatic or aromatic group [20]. The concentration-dependence of the spectral changes allows the binding affinities of the ligand to be estimated. A spectroscopic assay utilizing these properties has been routinely used to explore P450-ligand interactions in low-throughput format. Three major advantages of the assay that make it attractive for high-throughput applications are its simplicity, applicability to poorly characterized enzymes, and universality, as it can be easily adapted for analysis of any CYP obtainable in soluble form. The main drawbacks of the assay, however, are the relatively low-throughput and inherently low sensitivity of UV-vis absorption spectroscopy, which requires micromolar protein concentrations for screening. This in turn leads potentially to interference with test compound optical properties or solubility and to increased numbers of false-positive and false-negative hits [21]. Despite these disadvantages, a high throughput screening (HTS) assay based on the shift of the heme iron Soret band in response to small molecule binding has been successfully applied to CYP51's counterpart in *Mycobacterium tuberculosis* [22], ultimately resulting in identification of a potent *T. cruzi* inhibitor [14], [23] whose drug-like properties for Chagas Disease are currently being optimized.

The successful development of a structurally characterized, high expression recombinant *T. cruzi* CYP51 [24] has led to the establishment of an experimental platform for direct high-throughput exploration of the CYP51 active site capable of accommodating larger and more diverse libraries. Here we describe how a library of ∼104,000 small molecules was reduced to 185 structurally diverse positive hits with estimated K*_D_* values in the nanomolar range. Cross-validation of hits against *T. cruzi* parasites cultured in mammalian cells revealed 57 hits with anti-*T. cruzi* activity defined by EC_50_ values below 10 µM. While a region of structural similarity among the high affinity hits is dictated by the requirement for an aromatic heterocycle capable of coordinating to the heme iron, other parts of the molecules are not so constrained, allowing for unprecedented structural diversity among potential hits and opening new horizons for medicinal chemistry exploration of CYP51.

## Materials and Methods

### CYP51 target expression and purification

A previously constructed expression vector [24] was used to generate the recombinant *T. cruzi* CYP51 described in this work. The CYP51 utilized in the HTS screens had the first 21 residues upstream of K22 replaced with the fragment MAKKKKK and was His_6_-tag-engineered at the C-terminus. Protein was purified as described elsewhere [24] and was observed by means of 12% SDS-PAGE to be virtually homogeneous. Chromatographic fractions containing CYP51 in 20 mM potassium phosphate, pH 8.2, 10% glycerol, 0.5 mM EDTA, and 1 mM DTT and 200 mM NaCl were combined, concentrated and stored at −80°C. As expected, the enzyme solution displayed an absorbance peak at 420 nm, with a 449 nm peak appearing after reduction with sodium dithionite and bubbling with CO [24]. The concentration of functional P450 was determined from difference spectra using the extinction coefficient ε_450–490_ = 91000 M^−1^ cm^−1^ [25]. Purified CYP51 was shown to be stable under screening conditions at room temperature for up to 4 hours.

### High-throughput assay overview

**Figure 1:**
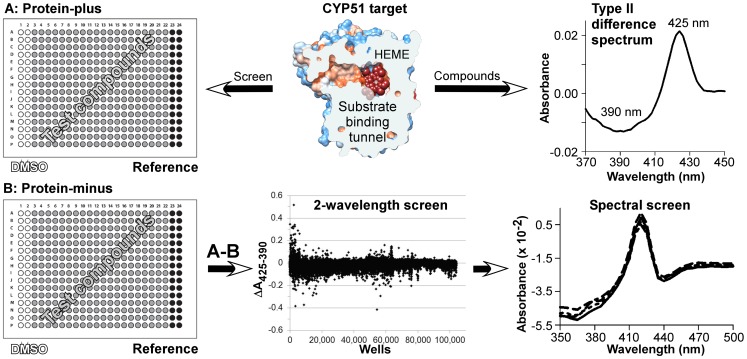
High throughput assay overview. *T. cruzi* CYP51 target is shown clipped by plane through the substrate binding tunnel (*top*, *center*). Type II difference spectrum is recorded for 1 µM CYP51 at saturated concentration of reference compound (*top*, *right*). Screen plates were prepared in duplicate, A and B, with target protein loaded only in A set (*left*). HTS scatter plot shows absorbance differences of test compounds (ΔA = A_425_−A_390_) measured using the two-wavelength detection mode; each point represents a single test compound (*bottom*, *center*). Overlapped spectra are shown for a sub-micromolar hit re-evaluated in spectral mode at four serial dilutions (*bottom*, *right*).

The compound library of the UCSF Small Molecule Discovery Center (SMDC), consisting of 103,986 diverse small molecules purchased from ChemBridge (San Diego, California), ChemDiv (San Diego, California) and SPECS (Delft, Netherlands) was screened against recombinant *T. cruzi* CYP51 in 384-well format using the protocol developed for *M. tuberculosis* CYP51 and described elsewhere [21]. The following modifications were introduced in the protocol to improve signal-to-noise ratio, throughput and reproducibility of measurements. First, the working protein concentration was increased to 2 µM and compounds were screened at 12.5 µM using two-wavelength detection. In this primary screen, plate reading was performed at 425 nm and 390 nm, corresponding to the maxima and minima of the difference spectra resulting from type II binding in the CYP51 active site (**Figure 1**). Type II inhibitors are characterized by their coordination bond to the heme iron, the single strongest interaction contributing to target affinity. Next, to account for organic solvent effects, protein background and the optical properties of test compounds, plates were prepared in duplicate, with target protein loaded in only a single set. After being read and processed separately, values generated from the protein-minus reference plate were subtracted from the corresponding values in the protein-plus test plate, yielding absorbance contributions due solely to the protein-compound interactions (**Figure 1**). Finally, to validate two-wavelength positive hits, four serial dilutions ranging from 2 µM to 12.5 µM were tested for a subset of positive hits using spectral detection mode, the spectra being recorded between 350 nm and 500 nm.

### Plate design, preparation and reading

All measurements were conducted in 100 mM potassium phosphate, pH 7.5, supplemented with 10% glycerol and, according to the plate layout shown in **Figure 1**, with dimethyl sulfoxide (DMSO) (columns 1 and 2), reference compound (columns 23–24) or individual test compounds (columns 3 through 22) at required concentrations. First, buffer alone (reference plates) or buffer containing 2 µM CYP51 (sample plates) was distributed in 80 µl aliquots using a Wellmate liquid dispenser (Thermo Fisher Matrix). Then test compounds, reference or DMSO were added in 1-µl aliquots using the automated 384-well head pipettor (Biomek FXP, Beckman Coulter) fitted with P20 tips (Fluotics Inc.). The compound {α-[(4-methylcyclohexyl)carbonyl amino]-N-4-pyridinyl-1H-indole-3-propanamide} purchased from ChemDiv (# C155-0123) (San Diego, California), referenced previously as LP-10 [14], [23], [26], was used as a type II reference. The compound and the reference stock solutions were prepared in DMSO at concentrations 80 times above working concentrations. DMSO alone was used as needed to hold DMSO concentration constant at 1.25% across the wells. Once prepared, plates were vortexed for 3 min, 800 rpm in a titer plate shaker (Lab-line Instrument, Inc., Pasadena, TX) and pulse-centrifuged for 10 sec, 1000 rpm (250 g) (Marathon 3000R, Fisher Scientific) to remove bubbles. Using a multimode plate reader (SpectraMax M5, Molecular Devices), the absorbance was recorded at 425 nm and 390 nm in two-wavelength mode, or at between 350 nm to 500 nm in 10 nm increments (16 measurements total/compound) in spectral mode, with 6 reads per well.

### Analysis of plate readings

**Figure 2:**
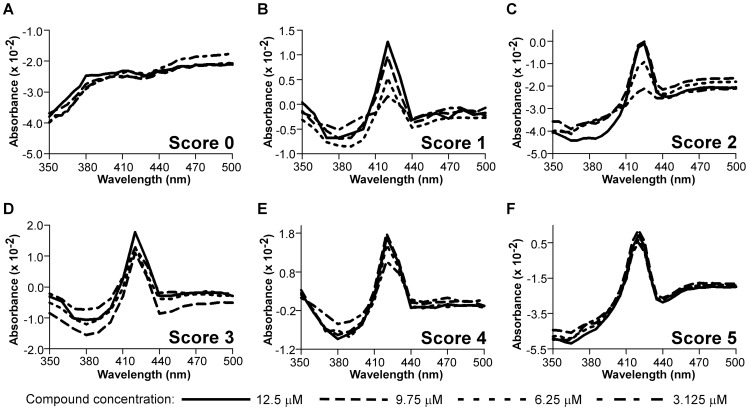
Scoring positive hits in spectral mode. Absorbance was monitored at four serial dilutions for each tested compound. (**A**) Lack of characteristic spectra-score 0; (**B**) concentration dependence with no spectra overlap – score 1; (**C**) any two spectra overlap-score 2; (**D**) any three spectra overlap-score 3; (**E**) three highest concentrations overlap-score 4; (**F**) all four spectra overlap-score 5.

Data were manipulated and analyzed using Microsoft Office Excel 2007. Values from each pair of test and reference plates were read and exported to the pre-programmed Excel template and processed using the algorithm shown in **Table S1**. First, each plate was processed separately to account for the effects of DMSO and protein background. In this step absorbance values for each individual wavelength were averaged across all 32 wells in columns 1 and 2. The resulting value was subtracted from the absorbance measured at the same wavelengths for each individual compound (columns 3 through 22) or reference (columns 23–24). Next, the corrected absorbance values in the reference plate (**Table S1B**) were subtracted from those of corresponding wells in the test plate (**Table S1A**), yielding the absorbance contributions at each wavelength solely due to protein-compound interactions. In two-wavelength mode, ΔA = A_425_−A_390_ was calculated for each compound and plotted against the corresponding well (**Figure 1**). Due to the Soret band shifting red, ΔA is a positive value for type II hits. In spectral mode, corrected absorbance values were directly plotted against wavelength. The resulting difference binding spectra were overlaid and inspected visually to eliminate false-positive hits and to rank the remaining compounds in the order of their affinities to the CYP51 target (**Figure 2**).

### Ranking positive hits in spectral mode

For ranking purposes, compounds were scored according to the appearance of their spectra. A score of 0 was assigned to false-positive compound hits that failed to demonstrate characteristic type II spectra (**Figure 2A**). The hits that showed concentration dependence with no overlapped spectra received a score of 1, while hits with any 2 or 3 overlapped spectra received scores of 2 and 3, respectively (**Figure 2B–D**). Compounds whose three highest concentration spectra overlapped received a score of 4 (**Figure 2E**). Finally, hits having all four spectra overlapped, including spectra for the highest and lowest concentrations, received a score of 5 in this system (**Figure 2F**).

### Low throughput UV-vis binding assay

Hit validation in the low throughput UV-vis binding assay was performed according to a previously published but somewhat modified protocol [14]. UV-vis absorption spectra were recorded on a Cary scanning spectrophotometer (Varian) in the 1-cm path length quartz cuvette at 23°C in 100 mM potassium phosphate buffer, pH 7.5, containing 10% glycerol, with CYP51 concentration of 0.5 µM. Compound solutions used for titration were prepared in DMSO at 100 µM and added in 2-µl aliquots to each 3 ml sample using a P2 pipetman (Gilson). Binding affinity of hits was estimated using the quadratic tight-binding equation [27]:


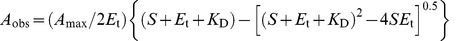
(1)

where A_obs_ is the absorption shift determined at any ligand concentration; A_max_ is the maximal absorption shift obtained at saturation; K*_D_* is dissociation constant for the inhibitor-enzyme complex; S is the ligand concentration; E_t_ is the total enzyme concentration.

### Mammalian cell culture and *T. cruzi* maintenance

Bovine embryo skeletal muscle cells [28] or mouse C2C12 myoblasts (ATCC#CRL-1772) used in the cross-validation assay, were cultivated respectively in RPMI 1640 medium supplemented with 5% horse serum and Dulbecco's Modified Eagle's Medium H-21 containing 4.5 g/l glucose (DMEM H-21) (Cell Culture Facility, UCSF), supplemented with 5% fetal bovine serum (FBS) (Sigma Chemical Co., St. Louis, MO, USA), 25 mM HEPES, 2 mM L-glutamine, 100 U/ml penicillin, 100 µg/ml streptomycin. *T. cruzi* CAI-72 trypomastigotes [28] were obtained from supernatant of infected cultures 4 to 7 days post-infection. Cultures were maintained at 37°C in an atmosphere of 5% CO_2_. To harvest parasites, the supernatant of infected cultures containing released trypomastigotes was collected and centrifuged at 600 rpm for 5 min to remove cell debris; the pellet was then discarded and the trypomastigotes remaining in the media were concentrated by spinning the culture tubes at 3300 rpm for 15 minutes. The parasite pellet was re-suspended in 2–5 ml of fresh media. Trypomastigote concentration was determined using a Neubauer hemocytometer.

### Hit cross-validation in *T. cruzi*-infected mammalian cells

Compound cross-validation in *T. cruzi* infected skeletal myoblasts (bovine embryo skeletal muscle or C2C12 lineage) was performed according to the protocol adapted from Engel and co-authors [29]. Compounds stored as 10 mM stocks in DMSO were freshly diluted immediately prior to use. Final concentrations of DMSO never exceeded 0.4%, which has been shown to be non-toxic both for parasites and mammalian cells. To prepare plates for the assay, skeletal myoblasts cultured in 75–150 cm^3^ flasks were washed once with phosphate buffered saline (PBS) and then incubated with 0.25% trypsin in PBS for 1–3 minutes for detachment. The trypsin solution was removed and myoblasts were released from the flask bottom. Fresh medium was added and cell concentration was determined using a Neubauer hemocytometer. Myoblasts in 50 µl of culture medium were transferred to the clear-bottom wells of black 96-well plates (Greiner Bio-One, #655090) at a density of 1500 cells/well and infected with 50 µl trypomastigote culture at 7500 parasites/well (1∶5 ratio). The cells were allowed to adhere and spread for 24 h in the incubator before the media was removed and 100 µl of fresh medium containing 0, 0.04, 0.12, 0.37, 1.10, 3.30 or 10 µM of test compound was added to each well, followed by 72 h incubation at 37°C in an atmosphere of 5% CO_2_. Posaconazole was used as a positive control at the same concentrations as the test compounds. The cells were then fixed by adding 100 µl/well of 8% formaldehyde in PBS, resulting in a 4% working concentration of formaldehyde. To stain the nucleus of the host cell and the nucleus and kinetoplast of parasites, cell membranes were first permeabilized by washing once with 100 µl of 0.1% Triton X-100 in PBS, then treated for 1 h with 0.2 µg/ml of the fluorescent DNA dye, 4,6-diamidino-2-phenylindole (DAPI), followed by one wash with PBS. The fixed plates were kept at 4°C and protected from light until images were acquired.

### Image acquisition and data analysis

**Figure 3:**
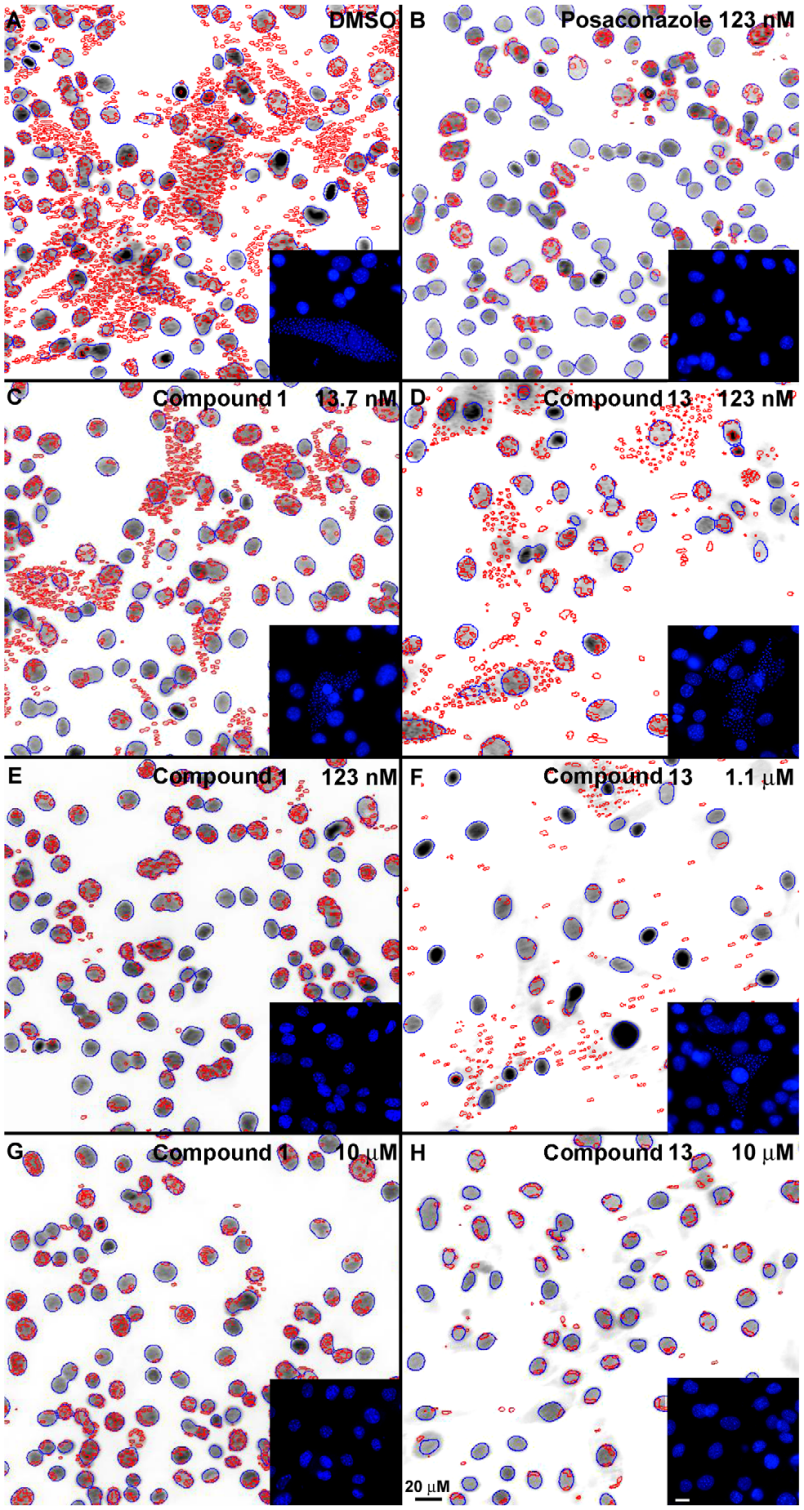
Cross-validation of hit compounds in intracellular *T. cruzi* amastigotes. Segmented images of DAPI-stained *T.cruzi*-infected myoblasts processed with image processing software are shown for cells cultured after 72 h treatment with Compounds **1** (**C**, **E**, **G**) or **13** (**D**, **F**, **H**) at indicated concentrations. Nuclei of host cells are highlighted in blue and nuclei and kinetoplasts of parasites are highlighted in red. The inserts show original DAPI staining in blue. Note the elimination of intracellular amastigotes at 0.123 µM and 10 µM of Compound **1** (**E**, **G**) and 10 µM of Compound **13** (**H**). In the negative control, DMSO-treated myoblasts show abundant parasitemia (**A**). Posaconazole was used as a positive control (**B**). Bars = 20 µM.

Plates were allowed to equilibrate to room temperature and images were acquired by the IN Cell Analyzer 2000 (GE Healthcare) with a 20× objective, 5 fields per well, with 350/50 nm and 460/40 nm fluorescent excitation and emission filters. In the absence of drug cytotoxicity, at least 800 cells were imaged and counted per well. The images were processed and analyzed by IN Cell Analyzer Developer 1.6 software. The size parameters used to segment host and parasite organelles were 125 µm^2^ for host nucleus, and 1–2 µm^2^ for parasite nucleus/kinetoplast. Since DAPI also strongly stains host heterochromatin regions that display as shapes resembling parasites (**Figure 3**), particles co-localized to host nuclei were excluded from quantification, while parasite aggregates near the host nucleus were counted as infected cells. Numbers of host cells and intracellular amastigotes were determined based on host cell and parasite nucleus and parasite kinetoplast quantification, providing a measure of growth inhibition during the first 72 h of post-infection treatment compared to untreated controls.

### Trypanocidal assay

Compounds cross-validated with low EC_50_ against *T. cruzi* infected cells were further evaluated to determine the “cidal” concentration at which host cells are effectively cured, using the protocol adapted from Engel et al 1998 [30]. To allow long term treatment and avoid culture overgrowth, C2C12 myoblasts were seeded at low density in 96 well plates (10^3^ cells/well) and cultured with DMEM H-21 medium supplemented with low FBS concentration (1%), 25 mM HEPES, 2 mM L-glutamine, 100 U/ml penicillin and 100 µg/ml streptomycin. The cells were infected with *T. cruzi* trypomastigote strain CAI/72 (5 parasites/cell) for 24 h and treated with 0, 0.04, 0.12, 0.37, 1.10, 3.30 or 10 µM test compounds in 100 µl of medium for 14 days. During the course of treatment, medium supplemented with test compounds was replaced every 72 h. After 14 days, drug pressure was withdrawn and incubation continued in the compound-free medium for another week, during which cultures were monitored for reappearance of *T. cruzi* trypomastigotes in the supernatant.

### Lipid analysis of *T. cruzi* amastigotes

To grow intracellular amastigotes for lipid analysis, C2C12 mouse skeletal myoblasts (4×10^6^ cells) cultured in 150 cm^2^ flasks as described above were infected with 80×10^6^ trypomastigotes for 24 h. The cultures were treated with test compounds 72 h post-infection at concentrations 5× below cidal to avoid completely killing the parasites. Posaconazole at 100 nM was used as a positive control. The cysteine protease cruzain inhibitor K777 [31] at 1.6 µM was used as a negative control to ensure that *T. cruzi* inhibition via other pathways did not affect composition of the membrane sterols. Cells were harvested 96 h post-infection and after 24 h of drug treatment. Cell detachment was initiated by brief treatment with 10 ml of 0.25% trypsin in PBS, followed promptly by replacement with 10 ml of fresh media containing FBS. Harvested cells were washed 3× with 25 ml of PBS to remove FBS and released trypomastigotes. The suspension of infected cells was then transferred to a glass tube and centrifuged at 2000 rpm for 5 min. After washing with PBS, 2 ml of chloroform-methanol solution (2∶1 ratio) was added to re-suspend the cell pellet. Organic solvents were then evaporated under N_2_ flow, allowing the pellet to dry.

For total lipid extraction, the dry cell pellet was treated with 2 ml chloroform for 24 h. Polar molecules were removed by washing the organic phase 3× with 3 ml water. The organic phase containing the sterols was subsequently dried on an evaporator under N_2_ flow. The lipids were re-suspended in 2 ml of chloroform-methanol (9∶1 ratio), washed again 3× with 3 ml water, dried under N_2_ flow and re-suspended in acetonitrile. After an additional wash 3× with 3 ml water, the acetonitrile was dried under an N_2_ stream. Extracted dry sterols were re-dissolved in hexane and derivatized with *N*,*N*-bis(trimethylsilyl)-2,2,2-trifluoroacetamide (BSTFA) (Pierce) by incubating at 37°C for at least 2 hours. Sterols as their trimethylsilyl (TMS) derivatives were analyzed by gas chromatography and mass spectrometry (GC-MS). GC-MS analysis and lipid identification were performed as described previously [14].

## Results

### Primary screening in two-wavelength mode

Given the large size of the library, we reasoned that use of the two-wavelength detection mode would increase the throughput potential of the assay above that of the multi-wavelength (spectral) mode previously described [21], [22]. Thus, in the primary screen, detection was carried out at 425 nm and 390 nm, corresponding to the maxima and minima of type II difference binding spectra, the difference in absorbance ΔA = A_425_−A_390_ being the discriminative criterion (**Figure 1**). The ΔA of the DMSO control averaged 0.000±0.003 (mean ± standard deviation), while ΔA of the reference was 0.043±0.004, resulting in the assay Z′-factor value of 0.58±0.04. Standard deviation was calculated for 528 measurements. The amplitude of the absorbance difference is directly proportional to the fraction of CYP51 target bound to the compound, with the maximal value of ∼0.04 corresponding to full displacement of the iron axial water ligand as a result of saturation of the active sites. 374 compounds (0.35% of the whole library) were outliers with ΔA>0.04. We speculate that these compounds fell into the outlier category because of spectral or solubility characteristics. Twenty of these outliers were selected for re-evaluation in spectral mode and were confirmed to lack characteristic absorbance features.

### Re-evaluation of two-wavelength hits in spectral screening mode

We identified false-positive hits in the two-wavelength pool by re-evaluating select compounds in spectral mode. 2,718 compounds with 0.016<ΔA<0.04 were first analyzed using Accelrys software (Accelrys, Inc., San Diego, California) for structural differences and similarities. Briefly, the fingerprint of each compound was determined using a proprietary extended-connectivity algorithm, EFCP_6, and folded to 256 bits for efficiency of comparison [32], [33]. A maximal dissimilarity [34] partitioning algorithm [35] employed the Tanimoto distance between these fingerprints to cluster the compounds into 30 groups. Similarly, 374 outliers with ΔA>0.04 were divided in 10 groups. Two representatives (one with maximum ΔA and another with median ΔA) were selected from each group, resulting in 80 compounds subjected to evaluation in full spectral mode. Evaluation was performed for four serial dilutions from 2 µM to 12 µM, followed by ranking of hits using the 0–5 scoring system (**Figure 2**). All 20 compounds selected from the outlier pool fell into the false-positive category (score 0). Among the remaining 60 compounds, 22 (36%) were validated as positive hits, including 7 compounds scored 4 or 5, with only one having all four spectra overlapped (score 5) (**Table S2**).

### Selection and re-evaluation of the 475 additional two-wavelength hits in spectral mode

A visual inspection of the first 22 hits shown to be positive in both two-wavelength and spectral modes confirmed that 17 possessed nitrogen-containing aromatic heterocycles capable of coordinating to the heme iron in the active site of CYP51 (**Table S2**). The remaining five contained aliphatic amine groups. Informatics analysis of the entire 104,000 compound library identified 6,643 compounds having heterocycle pharmacophores with an accessible nitrogen atom in their structures; 1,274 of these were identified in the two-wavelength primary screen as demonstrating absorbance differences in the range of positive hits. Strict enforcement of the Lipinski “rule of five” [36] narrowed the pool to 1,172 compounds. Hit scaffolds, defined as consisting of >2 rings and >3 members, were clustered by structural similarity and organized into a hierarchical tree using SARvision Plus 3.1 (Altoris, San Diego) software. Based on these criteria, 188 scaffold groups plus 155 singletons were identified. 320 compounds were picked from the scaffold groups and combined with the 155 singletons, yielding 475 compounds to be re-tested in the spectral mode and ranked using the 0–5 scoring system shown in **Figure 2**. Among these, only 21 compounds (4%) failed to demonstrate characteristic type II spectra, while the remaining 454 compounds showed the spectral characteristics of positive hits, including 134 hits which received a score of 4, and 44 hits with a score of 5, both values suggesting low nanomolar binding affinity as approximated by equation **(1)**. Combined with 7 previously identified hits, they constituted the 185 high affinity hits listed in the **Table S3**. The high target concentration dictated by the assay's sensitivity did not allow further discrimination of positive hits by their affinities in HTS format.

**Figure 4:**
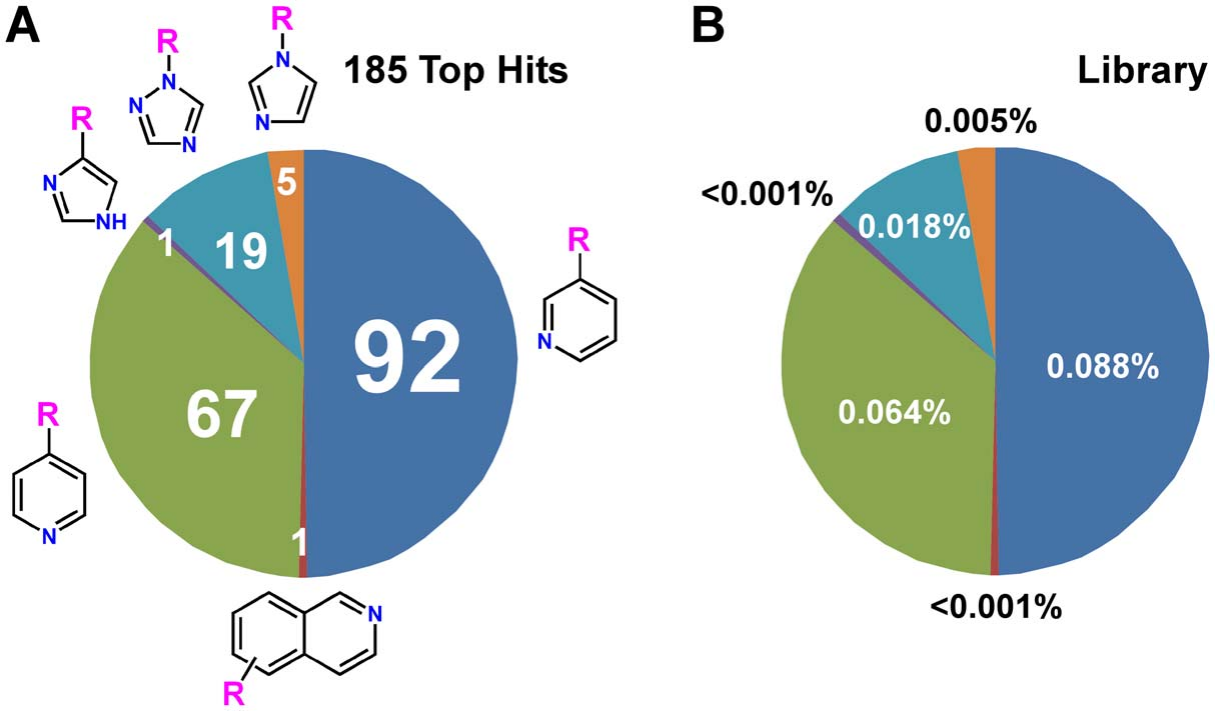
Nitrogen-containing aromatic heterocyclic pharmacophores. Distribution of nitrogen-containing aromatic heterocyclic pharmacophores among 185 positive hits with binding score 4 or 5 (**A**) echoes their frequency in the library (**B**). R represents diverse chemical structures as shown in **Table S3**.

Based on the structure of the putative heme Fe-coordinating modules, 185 hits can be divided in six unevenly populated categories (**Figure 4A**). The number of compounds populating each category varied from 1 to 92. This distribution reflects the frequency of each category in the library (**Figure 4B**), suggesting a lack of preference for the Fe-coordinating heterocycle as scored by this assay. Each of the 6 categories was further subdivided into clusters sharing more extensive structural similarities among the members (**Figure S1**). Some clusters had a higher number of *T. cruzi*-active hits (highlighted green in **Figure S1**) than others.

### Cross-validation of hits in *T. cruzi*-infected mammalian cells

The set of 475 compounds plus the 80 hits previously validated in spectral mode were cross-validated in *T. cruzi*-infected myoblast cells using an image-based medium throughput assay [29] (**Figure 3**). Posaconazole was used as a positive control with an EC_50_ of 1 nM. EC_50_ was determined as a concentration of compound corresponding to 50% inhibition of parasite endocytosis and intracellular growth, the calculation being based on the sigmoidal dose-response curve of compound-induced inhibition of the *T. cruzi* infection index (i.e., the percentage of *T. cruzi-*infected cells in culture multiplied by the mean number of parasites per infected cell).

**Figure 5:**
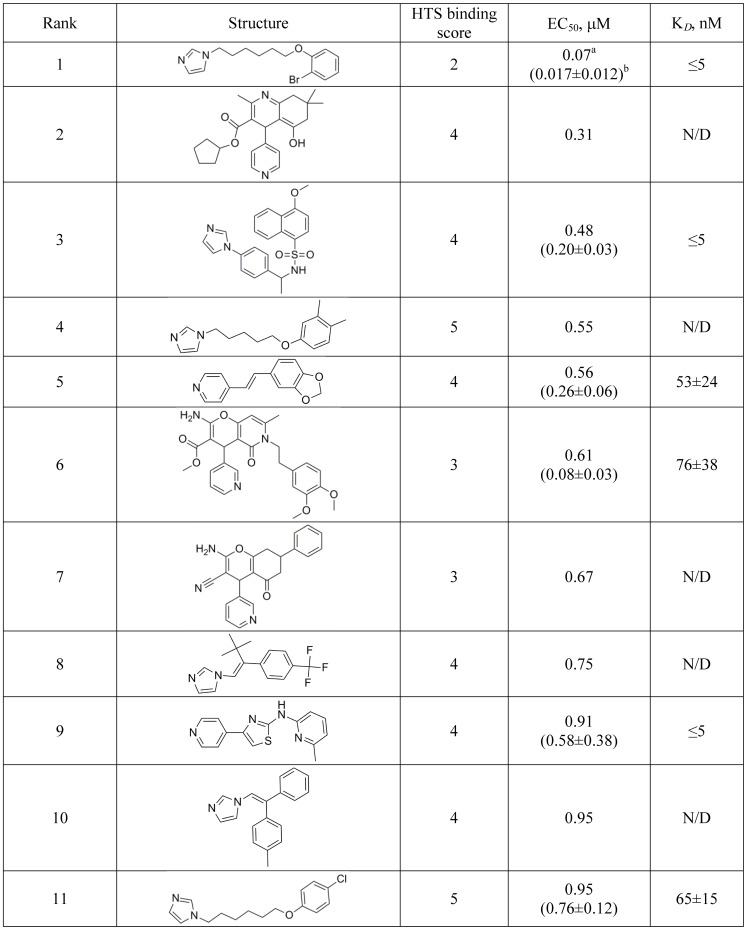
Sub-micromolar *T. cruzi* inhibitors. ^a^EC_50_ values obtained against *T. cruzi* parasites in HTS assay used to rank order the hits. ^b^In parenthesis are EC_50_ averaged from two independent cross-validation assays for individually repurchased hits.

Of the 497 cross-validated compounds, 57 exhibited anti-*T. cruzi* activity with EC_50_ below 10 µM; this group was notably enriched with CYP51 binding hit scores of 4 and 5 (36 of 57 total hits) (**Table 1**). The remaining hits (21 of 57 total hits, including the highest rated hit) had binding scores ranging from 1 to 3. The complete list of the 57 *T. cruzi*-active hits is shown in **Table S4**. A subset of eleven sub-micromolar hits is shown in **Figure 5**. Numbering of compounds in **Figure 5** and **Table S4** is according to their descending rank order. Both the 185-compound spectral pool (http://zinc.docking.org/users/lpodust/carts/185CYP51spectralHTShits) and the 57-compound *T. cruzi*-active pool (http://zinc.docking.org/users/lpodust/carts/57Tcruziactivehits) were uploaded to and made publicly available through the free online resource ZINC (http://zinc.docking.org) [37].

**Table 1:** Distribution of the 57 hits with EC_50_ ≤10 µM by binding scores.

Find: Symbols	Number of hits with EC_50_ ≤10 µM (57 total)
1	4
2	9
3	8
4	21
5	15

**Figure 6:**
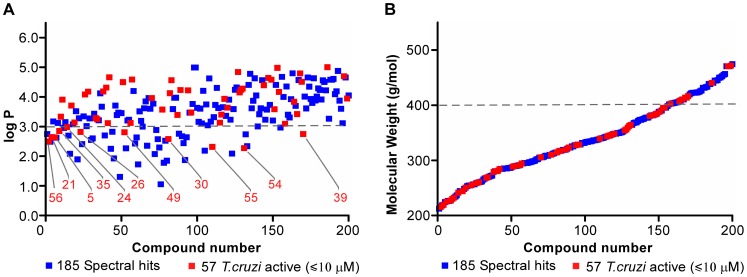
Comparative analysis of spectral (blue) *vs. T. cruzi*-active (red) hits. Distribution of the (**A**) logP and (**B**) molecular weight (MW) values.

Comparative analysis of both spectral and *T. cruzi*-active pools by logP and MW distributions showed good correlation between the two pools (**Figure 6**). A slight enrichment of hits with logP >3 in the *T. cruzi* active pool (82.5% in the *T. cruzi*-active pool *vs.* 78.9% in the pool of spectral hits) was observed (**Figure 6A**), with the molecular weight being rather evenly distributed across the range of 200–400 g/mol, with only a few hits crossing the 400 g/mol barrier (**Figure 6B**). Compound **5** with MW of 225 and calculated logP of 2.6 was the smallest molecule ranked among the top five *T. cruzi*-active hits.

**Figure 7:**
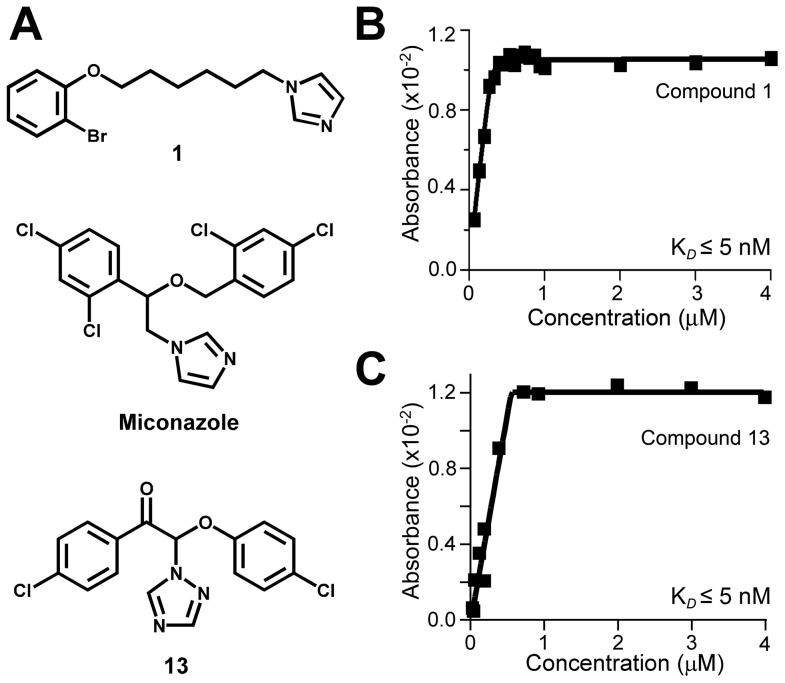
Hit validation in binding assay. (**A**) Hit compound structures. Compound 13 shows structural similarity to miconazole. Stoichiometric binding of **1** (**B**) and **13** (**C**) to CYP51 at 0.5 µM by UV-vis titration suggests low nanomolar binding affinity as approximated by quadratic tight-binding equation **(1)**.

Compound **1**, [6-(2-bromophenoxy)hexyl]imidazole, (MW of 323, logP of 4.2) was ranked first with an EC_50_ of 70 nM and HTS binding score of 2. Upon more rigorous validation in low throughput assays, Compound **1** was shown to have at least low nanomolar binding affinity to the target (**Figure 7**) and an EC_50_ of 17±12 nM averaged from the two independent assays against *T. cruzi* amastigotes cultured in mouse myoblasts (**Figure 3**). Trypanocidal concentration of Compound **1** was 40 nM in three independent experiments, which compares favorably to posaconazole (EC_50_ of 1 nM, trypanocidal at 0.6 nM).

### Re-mining data for sub-micromolar *T. cruzi* hits

**Figure 8:**
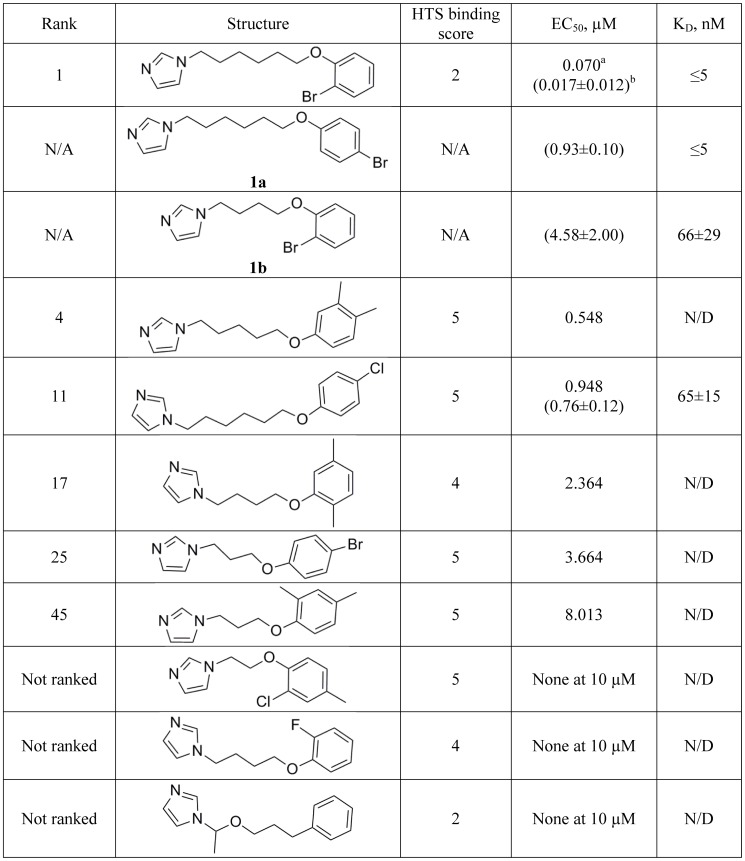
Highest rated hit compound cluster. ^a^EC_50_ values obtained against *T. cruzi* parasites in HTS assay used to rank order the hits. ^b^In parenthesis are EC_50_ averaged from two independent cross-validation assays for individually repurchased hits. N/A – not applicable; N/D – not determined.

Re-mining spectral screening data using the highly rated hits as molecular scaffolds, emulated a mini-SAR series providing insight to structure-activity relationships of the hits. For instance, Compound **1** was structurally distinct compared to azole antifungal drugs and the majority of positive hits identified in these studies. Two other highly ranked compounds, **4** and **11**, resembled Compound **1** (**Figure 5**). Altogether, seven structurally related hits were characterized by binding scores of 4 or 5, and two hits, including Compound **1**, by scores of 2 (**Figure 8**). In addition, two commercial analogs which were not part of the screened library, Compounds **1a** and **1b**, were tested. Compound **1a** differs by the position of the bromine atom in the bromophenoxy ring, whereas **1b** by the length of the alkyl linker between imidazole and bromophenoxy rings, both parameters seem essential for anti-*T. cruzi* activity (**Figure 8**). All variations presented in **Figure 8** are associated with reduced EC_50_, either due to drop in binding affinity to the target (e.g., Compound **1b** or Compound **11**), or possibly to other factors. Thus, relocation of the bromine atom from the meta- to the para-position of the bromophenoxy ring (Compound **1a**) results in an order of magnitude drop of anti-*T. cruzi* activity without detectable change in K*_D_*. However, an enzyme concentration of 0.5 µM defines the lower limit of the method's sensitivity at a K*_D_* of ∼5 nM, a hundredth of the target concentration, if a plateau in the titration curve is reached at the stoichiometric enzyme-ligand ratio (**Figure 7 B, C**). As differences in affinity between very tight-binding ligands are reflected in the sharpness of the titration curve, K*_D_* values recovered from fits to experimental data approximated by equation **(1)** are disproportionately sensitive to error in data points near the inflection point. Therefore, caution should be exercised when comparing the tight binding constants.

**Figure 9:**
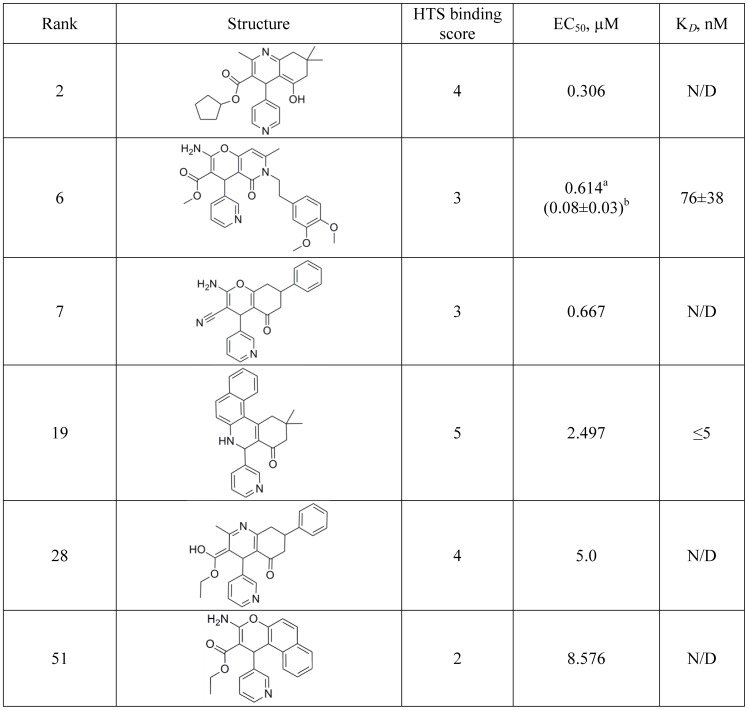
Second highest rated hit compound cluster. ^a^EC_50_ values obtained against *T.cruzi* parasites in HTS assay used to rank order the hits. ^b^In parenthesis is the EC_50_ obtained in validation assays for individually repurchased hits. N/A - not applicable; N/D - not determined.

Compounds **2**, **6** and **7**, highly rated in cross-validation, belong to their own molecular scaffold cluster, which includes 21 hits (**Table S5**). Hits rated highest in spectral mode (score 4 or 5) or in *T. cruzi* assay are summarized in (**Figure 9**). Compound **6**, methyl 2-amino-6-[2-(3,4-dimethoxyphenyl)ethyl]-7-methyl-5-oxo-4-pyridin-3-yl-5,6-dihydro-4H-pyrano[3,2-c]pyridine-3-carboxylate, was the only one from this cluster validated in low throughput format, including GC-MS analysis of sterol composition in treated amastigotes (not shown). Based on this validation, Compound **6** is the runner-up in the series of individually tested compounds, with estimated K*_D_* of 76 nM and EC_50_ of 80 nM. Further validation of Compound 2 and other representatives of this cluster are pending commercial availability of the compounds.

### Hit selection for manual validation

Two criteria – anti-*T. cruzi* activity and/or high screening score – were used to select commercially available compounds for more rigorous validation, including impact on the sterol biosynthesis in *T. cruzi* amastigotes cultured in mouse myoblasts. A search for compound vendors was conducted using the online resource ZINC [37]. Altogether, 32 hit compounds were acquired for validation in manual low-throughput assays, including eight *T. cruzi*-active hits: Compounds **1**, **3**, **5**, **6**, **9**, **11**, **13** and **15** (**Table S6**). Binding could not be confirmed for two of the acquired compounds due to compound precipitation under assay conditions. The other repurchased compounds bound CYP51 with one-to-one stoichiometry at 0.5 µM protein concentration (**Figure 7**), suggesting at least low nanomolar binding affinity as extrapolated from equation **(1)**. Low EC_50_ values were confirmed for all tested *T. cruzi*–active hits (**Figure 5**).

### GC-MS analysis of membrane sterols

**Figure 10:**
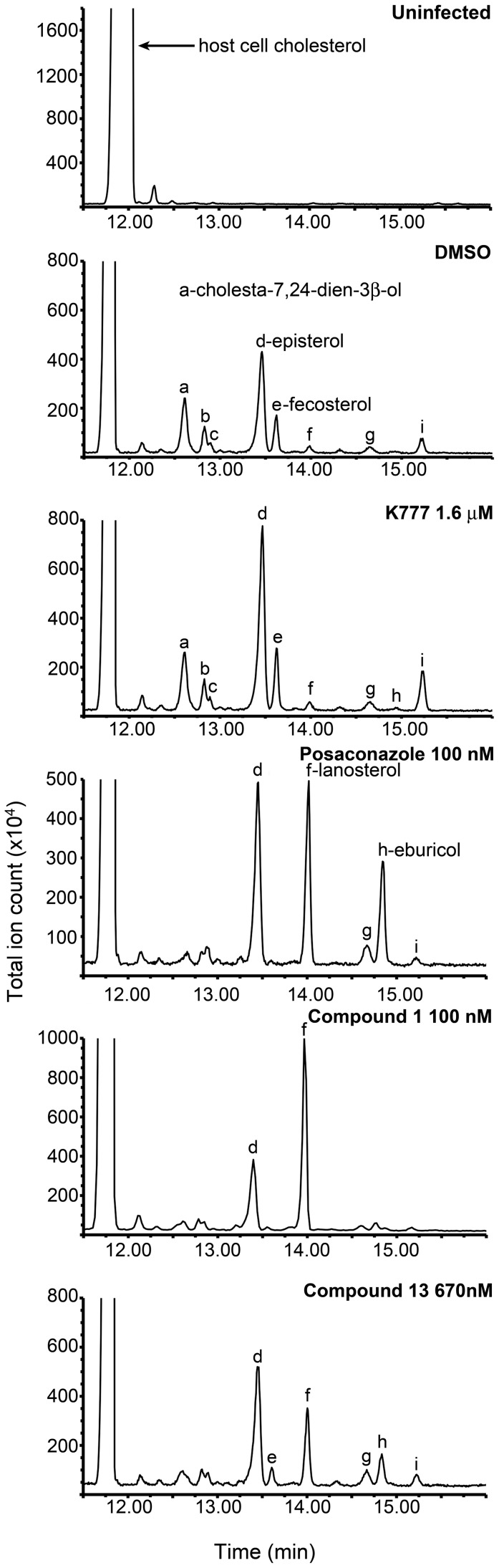
GC-MS analysis of the *T. cruzi* lipid extracts treated with the CYP51 inhibitors. DMSO and K777 were used as negative controls; posaconazole served as a positive control. Uninfected host cell panel (top) demonstrates that chromatographic peaks labeled with small-case letters from **a** to **i** are of *T. cruzi* origin, corresponding to **a** - cholesta-7,24-dien-3β-ol, [M]^•+^ = m/z 454; **b** - cholesta-8,24-dien-3β-ol (zymosterol), [M]^•+^ = m/z 470; **c** - 24-methyl-7-en-cholesta-en-3β-ol, [M]^•+^ = m/z 472; **d** - ergosta-7,24-diene-3β-ol (episterol), [M]^•+^ = m/z 470; **e** - ergosta-8,24-diene-3β-ol (fecosterol), [M]^•+^ = m/z 470; **f** - lanosterol, [M]^•+^ = m/z 498; **g** - 4-methylepisterol, [M]^•+^ = m/z 484; **h** - eburicol, [M]^•+^ = m/z 512; **i** - 24-ethyl-7,24(24′)-en-cholesta-dien-3β-ol, [M]^•+^ = m/z 484. Cholesterol is the only peak originating from host cells. Increase in the CYP51 substrates lanosterol (**f**) and eburicol (**h**) is due to inhibition of *T. cruzi* CYP51, as is the decline in the downstream major products episterol (**d**), fecosterol (**e**), cholesta-7,24-dien-3β-ol (**a**) and the minor products 4-methyl-episterol (**g**) and 24-ethyl-7,24(24′)en-cholestadien-3β-ol (**i**).

Inhibition of sterol biosynthesis by GC-MS analysis was confirmed for all individually acquired hits. The major sterol observed in untreated amastigotes was episterol (peak **d** in **Figure 10**), followed by approximately equal amounts of fecosterol (**e**) and cholesta-7,24-dien-3β-ol (peak **a**). This sterol composition is consistent with our previously published work [14] but differs from Liendo and co-authors [5], where 24-methyl-7-en-cholesta-en-3β-ol (peak **c** in **Figure 10**) was identified as a main sterol of intracellular amastigotes. The most straightforward explanation for these differences would be the differences in the lipid extraction protocols used in this work or published elsewhere [38]. As a result of CYP51 inhibition, two 14-methylated precursors, lanosterol (**f**) and eburicol (**h**) dominated the GC-MS traces of the strongest CYP51 inhibitors posaconazole and Compound **1**, and were present in amounts comparable to those of 14-demethylated products for the less potent Compound **13** (**Figure 10**). Interestingly, fecosterol (**e**) was completely inhibited by low concentrations of posaconazole and Compound **1**, whereas episterol (**d**) persisted. This observation seems difficult to explain in light of the *T. cruzi* sterol pathway reported elsewhere [5], in which fecosterol precedes episterol in the reaction chain.

### Cheminformatic analysis of hits

Given an unexpectedly large number of structurally diverse hits, we used an algorithm implemented in the online cheminformatic research tool SEA (Similarity Ensemble Approach) (http://sea.bkslab.org/) [39] to relate CYP51 to other pharmacologic targets by ligand topology. In SEA, the similarity of protein targets is established and scored based on the similarity of their ligands using the probability of a random match, or expectation E-value, as a scoring function that relates lower E-values to more statistically significant ligand similarities. Results from probing the molecule databases implemented in SEA [39] with the 57 *T. cruzi*-active hits, either individually or as a pool, indicated CYP51's similarity to other P450 drug targets, including thromboxane synthase (CYP5), fatty acid ω-hydroxylases (CYP4), 17α-hydroxylase/17,20-lyase (CYP17), and aromatase (CYP19). These enzymes have been targeted by the pharmaceutical industry for cardiovascular disease [40], metabolic disorders of lipid metabolism and inflammation [41], prostate cancer [42], and estrogen receptor-positive breast cancer [43], respectively. Unexpectedly, these pharmacologic targets scored substantially higher than sterol 14α-demethylase (CYP51) itself, giving credence to the idea that molecules directed against these targets, including drugs that have entered the clinic, could be examined for potency against *T. cruzi* CYP51.

Another surprise revealed by cheminformatic searching in the SEA databases is the striking resemblance of the highest rated hit to inhibitors of glutaminyl-peptide cyclotransferase, an enzyme unrelated to CYP family either by sequence or structure. This enzyme catalyzes the formation of N-terminal pyroglutamic acid whose biological function is either to mediate peptide hormone interaction with receptors, or to stabilize proteins and peptides against N-terminal degradation [44]. Human glutaminyl-cyclase which catalyzes pyroglutamic acid formation at the N-terminus of amyloid peptides, is potentially involved in the development and progression of neurodegenerative disorders [44].

## Discussion

**Figure 11:**
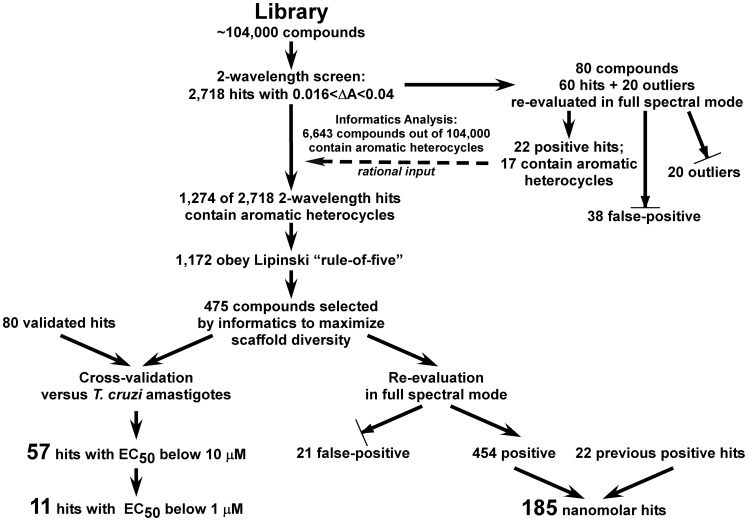
Flowchart diagram of screening and validation steps.

We designed an HTS assay to identify compounds capable of coordinating to the heme iron of CYP51. Following target-based screening and the further validation steps summarized in **Figure 11**, we identified 185 high affinity hits and 57 partially overlapping hits with anti-*T. cruzi* activity, including a dozen sub-micromolar inhibitors of *T. cruzi* infection in mammalian cells. Hits identified in the primary two-wavelength screen were evaluated by cheminformatic analysis, spectral screening and cross-validation against *T. cruzi* amastigotes. Informatics feedback served to maximize scaffold diversity and reduce the number of compounds subject to re-evaluation in the more laborious spectral mode. As a result, the two pools, of 185 and 57 hits, were structurally diverse with respect to the number, size and arrangement of rings, as well as the nature of the linkers and substituting groups constituting the compound frameworks (**Tables S3 and S4**). In summary, although two-wavelength mode is more efficient in terms of throughput, it generated >60% false-positive hits. Although more time-consuming to read, analyze and tabulate, the spectral mode is a more comprehensive methodology than the two-wavelength mode in terms of efficiently filtering and ranking true positive hits. Overall, based on validation tests for representative compounds, we expect the set of hits identified in this work to contain a high percentage of true positive hits and to be a valuable resource for further development of Chagas Disease applications.

At the high hit rate we observed, we were unlikely to miss important chemotypes by excluding 102 compounds violating Lipinski rule of five from validation in the full spectral mode and cross-validation in the *T. cruzi* assay. The process of optimization generally yields larger and more hydrophobic compounds, and thus it is industry practice to focus on smaller “lead-like” compounds to allow MW and logP to increase during lead development [45]. On the other hand, a rationale for looking at hydrophobic hits in more detail is justified by the fact that the CYP51 inhibitors itraconazole and posaconazole do not comply with this rule. The potency of posaconazole in the treatment of invasive fungal infections is attributed to accumulation of drugs in the tissues of internal solid organs rather than to free levels of drug in plasma [46], [47]. In isolated cells and solid tissues, both posaconazole and itraconazole are mostly concentrated in cellular membranes, which are also the site of their molecular target, CYP51 [48]. Given that the potent *in vivo* activity of posaconazole and other novel CYP51 inhibitors is attributed to their special pharmacokinetic properties, such as large volumes of distribution and long terminal half-life [49], [50], it may be worth revisiting the 102 hits initially excluded by the Lipinski criteria.

The *T. cruzi*–active pool contained both high affinity hits (score 4 or 5) and those with lower binding scores (**Table 1**), suggesting that low score compounds might have been acting via alternative mechanisms. To test this possibility, the binding and activity of the highest rated hit, Compound **1**, was confirmed in manual assays. Compound **1**, had activity an order of magnitude higher than any other hit identified in these studies (**Figure 5**). At 100 nM concentration, Compound **1** suppressed sterol biosynthesis in *T. cruzi* amastigotes more efficiently than posaconazole (**Figure 10**), thus becoming a candidate for further evaluation in an animal model of infection. Cheminformatic searching in the SEA databases [39] revealed a significant resemblance of the top hit to inhibitors of P450 drug targets involved in eicosanoid synthesis and fatty acid metabolism. For example, thromboxane-A synthase was highly ranked with regard to the top hit and the structurally related sub-micromolar hits **4** and **11**, as were the fatty acid ω-hydroxylases of the CYP4 family. Surprisingly, the lowest E-values were obtained for glutaminyl-peptide cyclotransferase, which is unrelated to CYP family by sequence or structure. While the ability of drugs to bind to unintended targets in the human host can lead to undesirable side effects, the same binding to a target in a pathogen infecting a human can cure infection. In the context of a deficit of drugs for neglected diseases, the unexpected promiscuity of CYP51 encountered in this work could be utilized in a “piggy-back” strategy [51], [52] for identifying a chemical starting point for anti-parasitic therapy development, while structure-activity relationships derived from parasite assays could lead to disease-specific clinical candidates [53]–[56].

The structural motif present in the cohort of antifungal azole drugs was apparent in both pools. For instance, hit 1, 2-(4-chlorophenoxy)-1-(4-chlorophenyl)-2-(1H-1,2,4-triazol-1-yl)ethanone (binding score 5) (Compound **13**), which ranked thirteenth in the anti-*T. cruzi* activity assay, resembled miconazole (**Figure 7A**), one of the first broad-spectrum antimycotic agents and the conazole progenitor of orally active antifungal agents such as ketoconazole, itraconazole, posaconazole, fluconazole and voriconazole [15]. Manual UV-vis titration confirmed that Compound **13** binds and saturates CYP51 at equimolar 0.5 µM concentrations, suggesting at least low nanomolar binding affinity (**Figure 7C**). According to the SciFinder online resource, compounds with close structural similarities to Compound **13** were patented in the 1970's as potent fungicides. To confirm a relationship between this structural motif and biological activity, we extracted membrane sterols from *T. cruzi* amastigotes isolated from infected mouse myoblasts treated with Compound **13**. Consistent with the inhibition of CYP51, GC-MS analysis showed an increase in lanosterol and eburicol precursors and a concomitant decline in episterol, fecosterol and other 14-demethylated intermediates (**Figure 10**). Compound **13** was effective against *T. cruzi* amastigotes with an EC_50_ of 1.3 µM and was trypanocidal at 2.5 µM.

The shape of the posaconazole molecule defines its binding mode to CYP51. Its long “tail” group extends into the substrate-binding tunnel [24], [57]. In the mouth of the tunnel, posaconazole adopts alternative conformations and makes multiple points of contact with amino acid residues on the protein surface [24]. Extensive interactions of posaconazole with catalytically non-essential residues in CYP51 are strikingly consistent with the pattern of drug resistance in fungi. The points of posaconazole contact are mutation hot spots in azole-resistant isolates of the pathogenic fungi *Aspergillus fumigatus* and *Candida albicans* [24]. Some of these mutations confer cross-resistance to all azole drugs [58]–[61]. The spread of drug resistance due to mutation of the target in response to therapy might be avoided in *T. cruzi* if the binding properties of new inhibitors were improved using target structure as a guide. This concept has been successfully used for a panel of 9 FDA-approved HIV protease inhibitors developed with extensive use of structure-based drug design [62]. In order to stay within a substrate envelope and focus hit-to-lead optimization chemistry on main-chain interactions, we are pursuing co-crystallization of low molecular weight hits with CYP51 for x-ray structure analysis. Compared to the larger posaconazole (MW 700 g/mol), the molecular weight of hits active against *T. cruzi* ranged from 215 to 480, with the majority (39 of 57) below 360; Compounds **1** and **13** are 323 and 348 g/mol, respectively. Furthermore, with its “fragment-like” characteristics [63] of the lowest molecular weight and logP among the sub-micromolar *T. cruzi* hits, Compound **5** has good prospects in hit-to-lead optimization, as does one of the smallest high affinity spectral hits, 5,8-dibromoisoquinoline (Compound **45** in **Table S3**; MW 287 g/mol), consisting only of an iron-coordinating heterocyclic module.

**In conclusion**, diversification of leads for CYP51 inhibitors should offer the medicinal chemist new choices in terms of chemical accessibility and prospects for lead optimization. Multiple leads lower the risk of drug attrition in the case of undesirable ADMET properties and also cover a patent-free space, one of the keys to the successful anti-Chagasic therapy of the future. In the course of this work we have (**i**) further developed a UV-based high throughput screening methodology for P450 enzymes; (**ii**) diversified the chemical scaffold space for CYP51 inhibitors; (**iii**) identified a potent *T. cruzi* inhibitor and a diverse array of low molecular weight hits with high affinity to CYP51 for hit-to-lead optimization; (**iv**) related CYP51 to other pharmacologic targets by computational ligand chemistry. This effort has allowed us to identify molecules already produced by pharmaceutical companies for future experimental testing against *T. cruzi*.

## Supporting Information

Figure S1 **Clustering of the 185 hits validated with the binding score 4 or 5.**
(PDF)

Table S1 **Analysis of plate readings.**
(DOCX)

Table S2 **First 22 hits validated positive both in two-wavelength and spectral screening modes.**
(DOCX)

Table S3 **185 hits validated with binding score 4 or 5.**
(DOCX)

Table S4 **57** ***T. cruzi*** **-active hits in descending rank order.**
(DOCX)

Table S5 **Second highest rated hit compound cluster.**
(DOCX)

Table S6 **32 individually validated hits.**
(DOCX)

Alternative Language Abstract S1 **Manuscript Abstract in Portuguese.**
(DOC)
