# Supplementary material for: Diverse Inhibitor Chemotypes Targeting Trypanosoma cruzi CYP51
Source: PLoS Negl Trop Dis. 2012 Jul 31;6(7):e1736. doi: 10.1371/journal.pntd.0001736 (PMC3409115; doi:10.1371/journal.pntd.0001736)
Supplement: Figure S1 — Clustering of the 185 hits validated with the binding score 4 or 5. (PDF) [file pntd.0001736.s001.pdf]

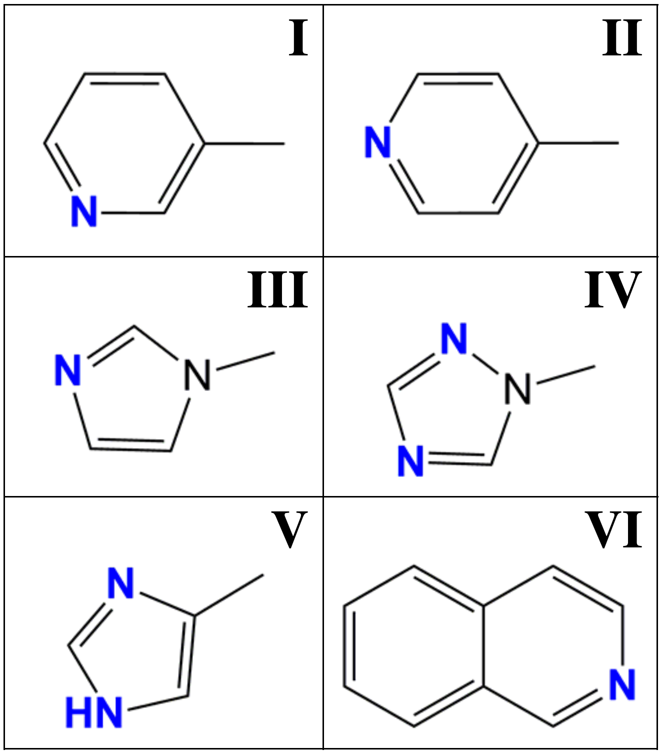

Figure S1. Clustering of the 185 hits validated with the binding scores 4 or 5. Each Fe-coordinating module is linked to the clusters of hits having in common a substructure highlighted in magenta. Aromatic nitrogen atoms capable of coordinating to the heme Fe are highlighted in blue. Representatives of different groups individually validated in low throughput assays are highlighted in yellow. *T. cruzi* active compounds are highlighted in green.

Cluster I
I-1 (21)

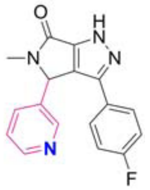	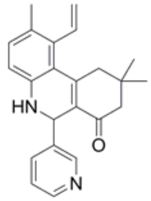	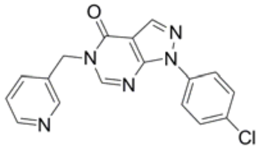	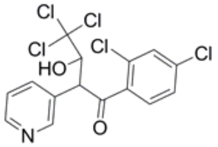
(5)	(5) EC$_{50}$ 2.497 μM	(5)	(5) EC$_{50}$ 6.873 μM
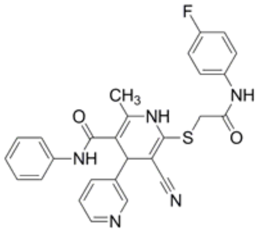	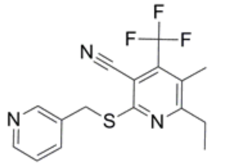	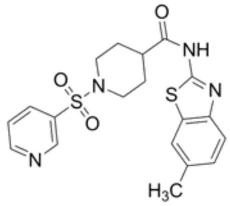	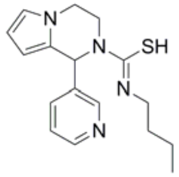
(5)	(5)	(4) EC$_{50}$ 7.64 μM	(4) EC$_{50}$ 6.335 μM
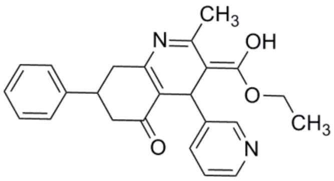	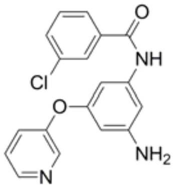	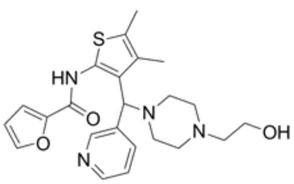	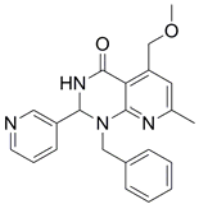
(4) EC$_{50}$ 5.0 μM	(4)	(4)	(4)
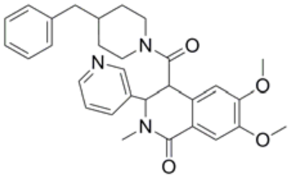	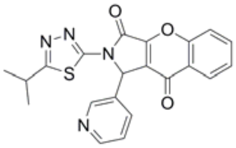	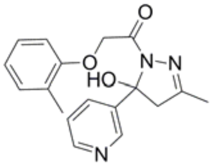	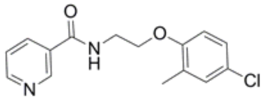
(4)	(4)	(4)	(4)
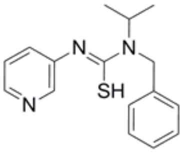	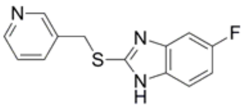	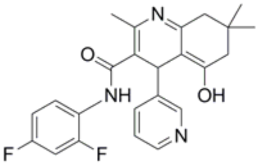	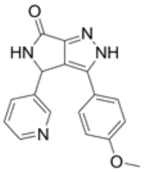
(4)	(4)	(4)	(4)
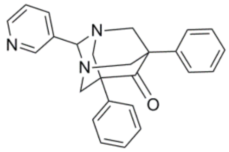			
(4)			

I-2 (26)

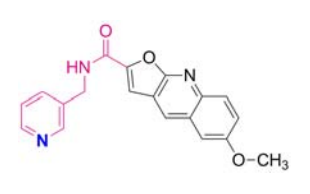	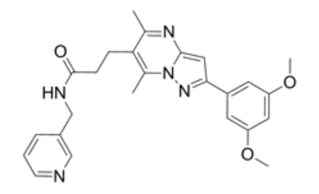	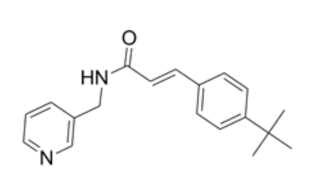	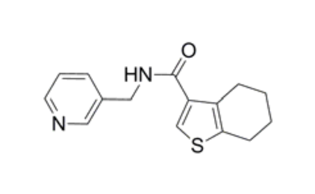
(5)	(5)	(5)	(5)
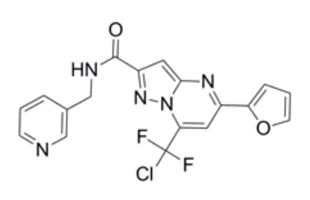	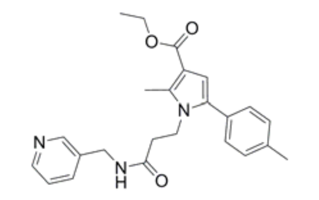	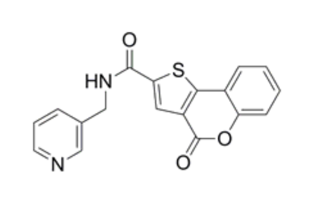	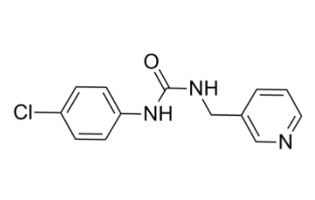
(5)	(5)	(5)	(5)
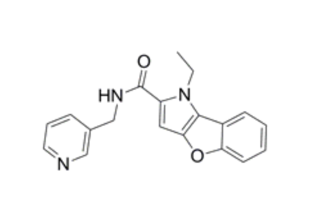	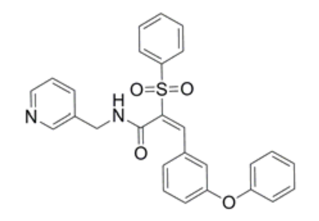	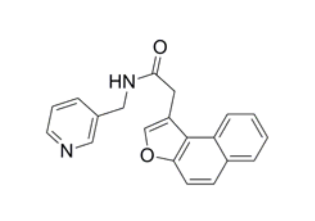	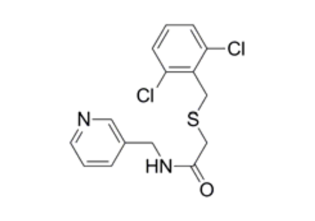
(4) EC_{50} 2.2 μM	(4) EC_{50} 8.993 μM	(4) EC_{50} 7.928 μM	(4)
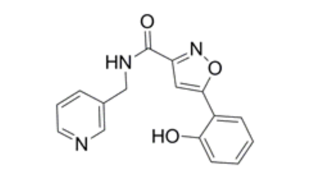	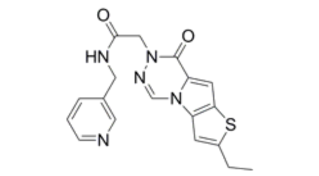	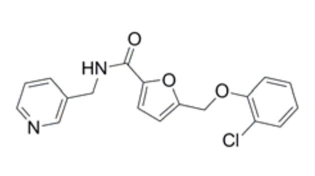	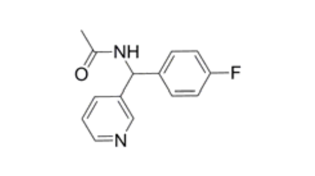
(4)	(4)	(4)	(4)
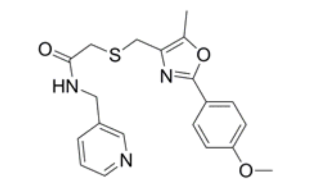	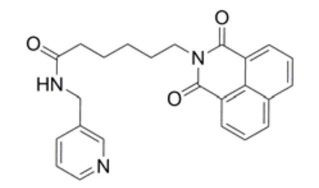	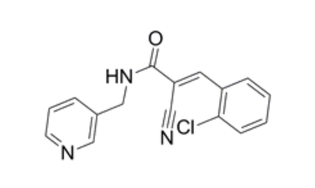	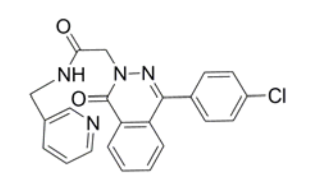
(4)	(4)	(4)	(4)
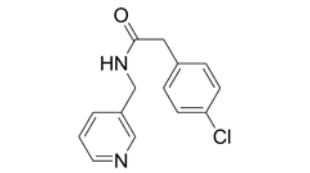	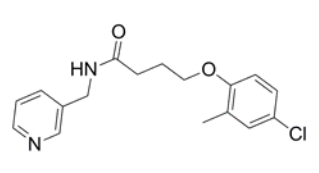	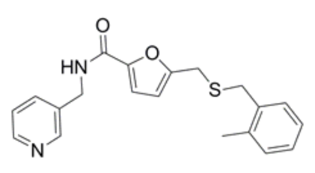	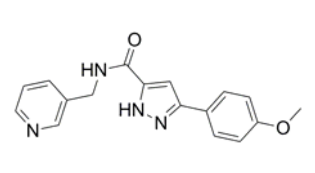
(4)	(4)	(4)	(4)
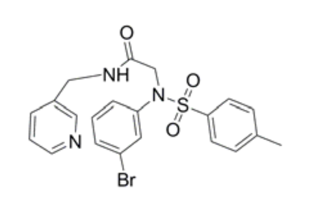	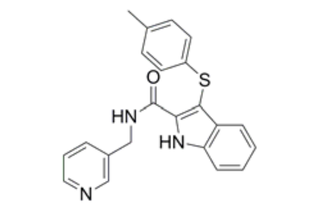		
(4)	(4)		

I-3 (11)

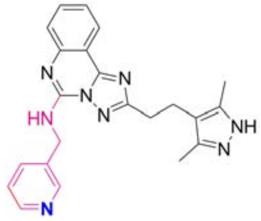	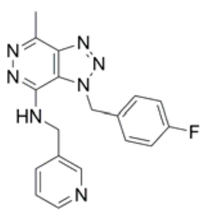	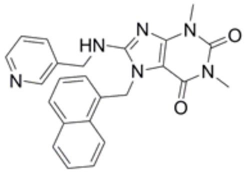	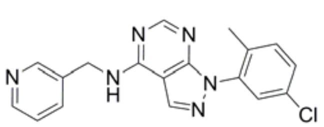
(5)	(5)	(4)	(4)
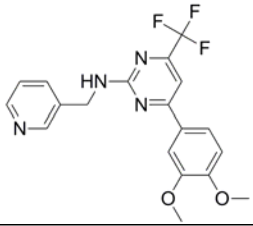	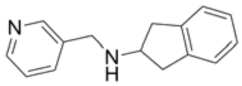	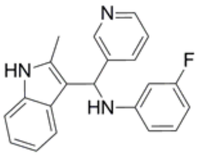	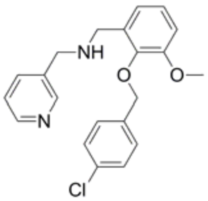
(4)	(5)	(4)	(4)
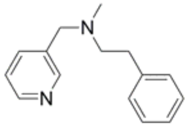	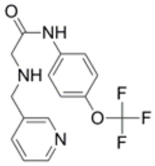	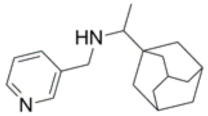	
(4)	(4)	(4)	

I-4 (5)

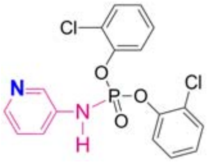	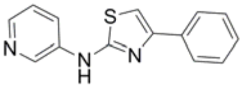	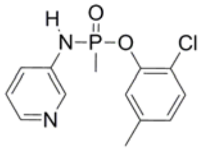	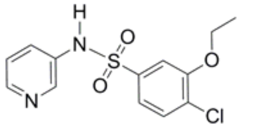
(5) EC$_{50}$ 7.731 μM	(4)	(4)	(4)
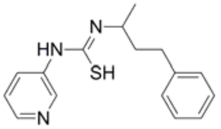			
(4)			

I-5 (5)

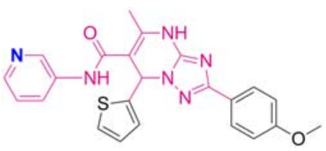	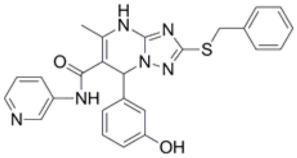	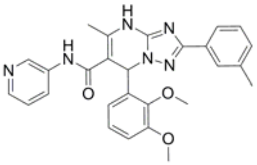	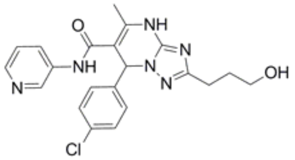
(4)	(4)	(4)	(4)
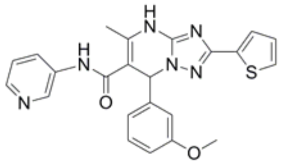			
(4)			

I-6 (5)

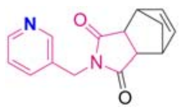	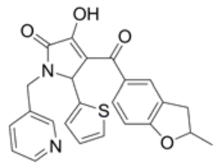	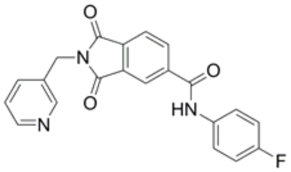	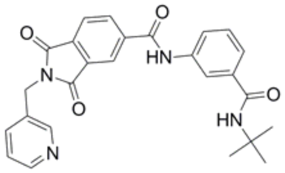
(5)	(4)	(4)	(4)
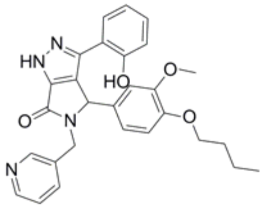			
(4)			

I-7 (4)

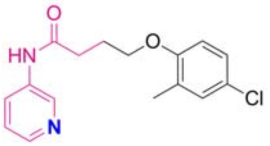	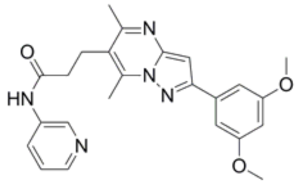	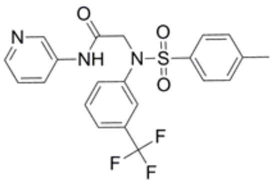	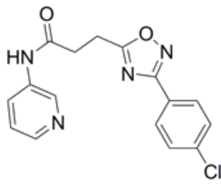
(4)	(4)	(4)	(4)

I-8 (3)

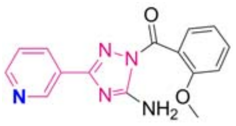	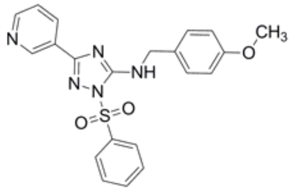	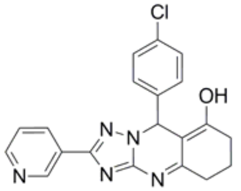
(4)	(4)	(4)

I-9 (3)

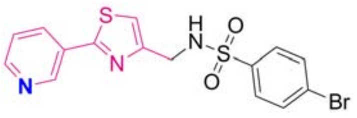	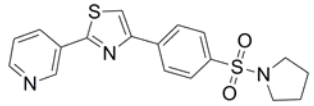	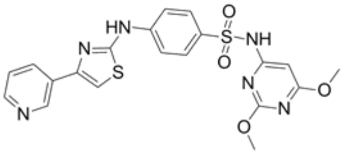
(5) EC_{50} 6.504 μM	(4)	(4)

I-10 (3)

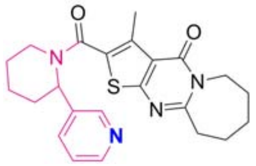	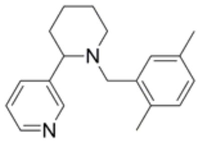	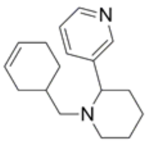
(4)	(4) EC_{50} 8.238 μM	(4)

I-11 (3)

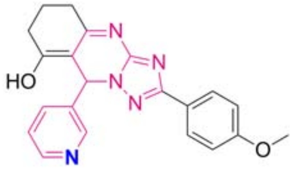	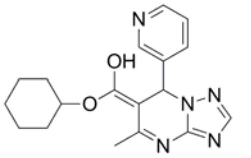	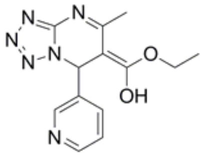
(5)	(4) EC_{50} 9.54 μM	(4)

I-12 (3)

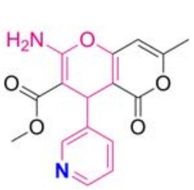	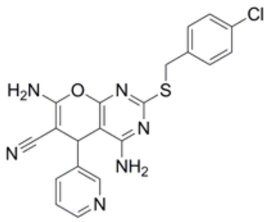	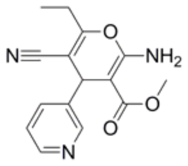
(4)	(4)	(4)

Cluster II

II-1 (22)

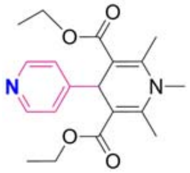	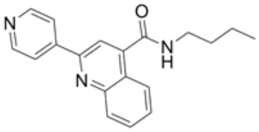	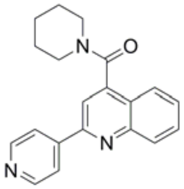	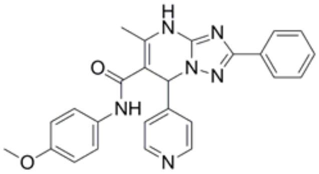
(5) EC$_{50}$ 5.179 μM	(5) EC$_{50}$ 8.401 μM	(5)	(5)
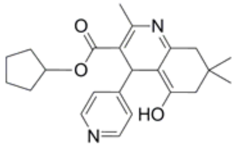	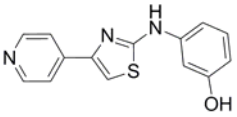	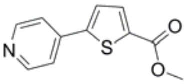	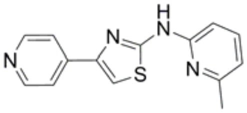
(4) EC$_{50}$ 0.306 μM	(4) EC$_{50}$ 7.326 μM	(4) EC$_{50}$ 2.738 μM	(4) EC$_{50}$ 0.914 μM
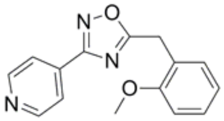	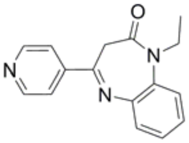	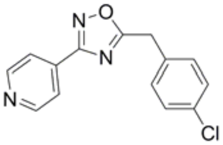	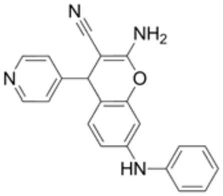
(4)	(4)	(4)	(4)
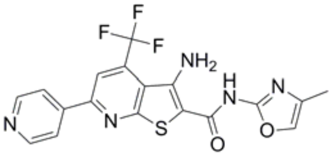	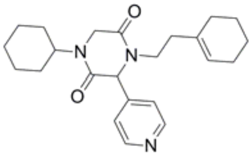	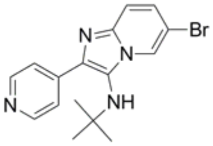	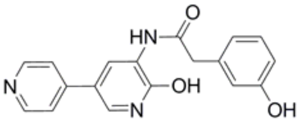
(4)	(4)	(4)	(4)
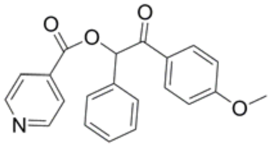	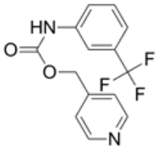	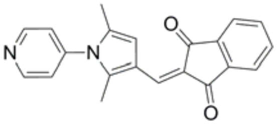	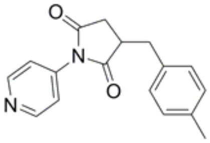
(4)	(4)	(4)	(4)
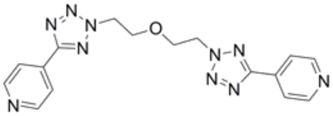	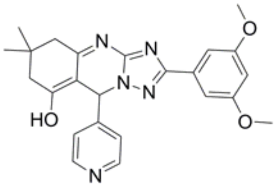		
(4)	(4)		

II-2 (9)

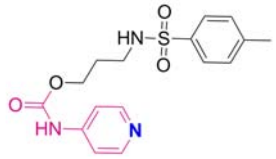	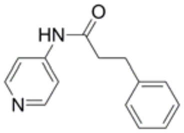	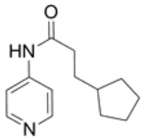	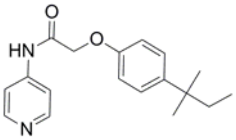
(5)	(5)	(4)	(4)
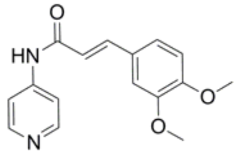	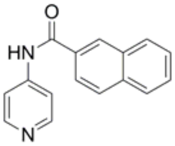	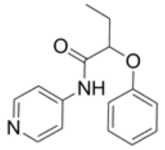	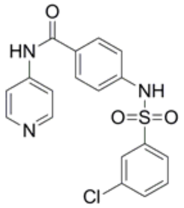
(4)	(4)	(4)	(4)
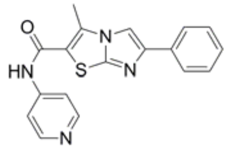			
(4)			

II-3 (8)

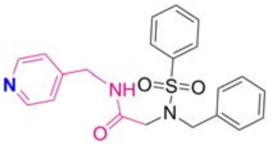	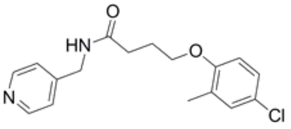	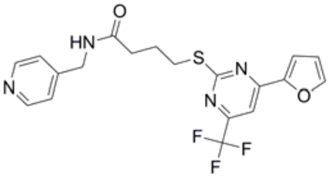	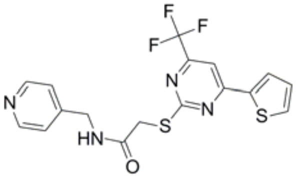
(5)	(4)	(4)	(4)
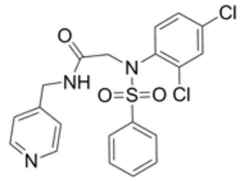	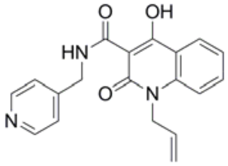	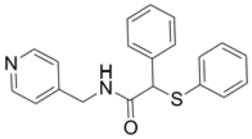	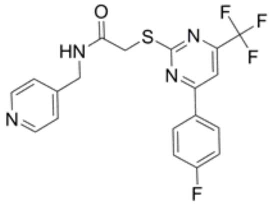
(4)	(4)	(4)	(4)

II-4 (5)

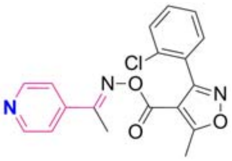	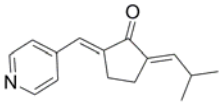	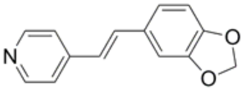	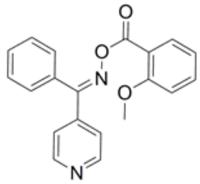
(5) EC_{50} 6.779 µM	(4) EC_{50} 7.148 µM	(4) EC_{50} 0.564 µM	(4)
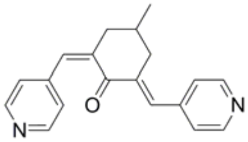			
(4)			

II-5 (5)

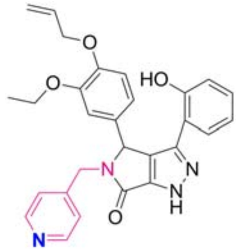	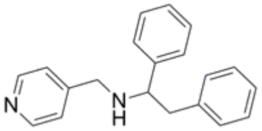	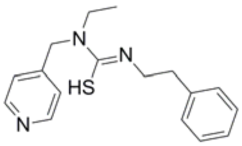	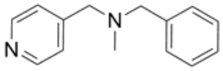
(5)	(5) EC_{50} 4.822 µM	(5)	(4)
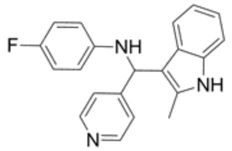			
(4)			

II-6 (5)

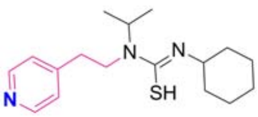	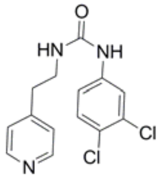	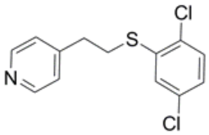	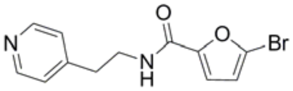
(5)	(4) EC_{50} 2.548 µM	(4) EC_{50} 7.413 µM	(4)
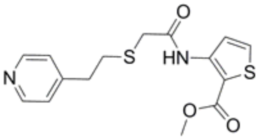			
(4)			

II-7 (4)

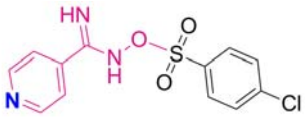	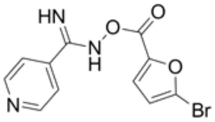	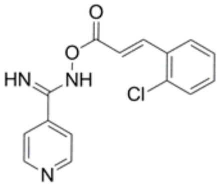	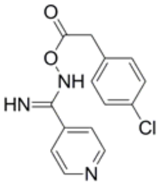
(5)	(4)	(4)	(4)

II-8 (3)

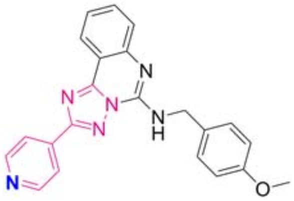	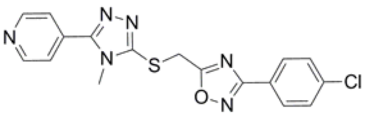	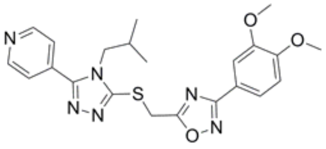
(5)EC_{50} 1.929 µM	(5) EC_{50} 7.674 µM	(4)

II-9 (3)

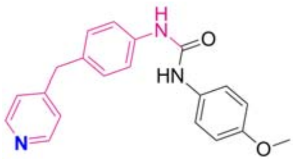	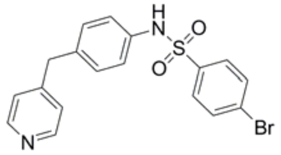	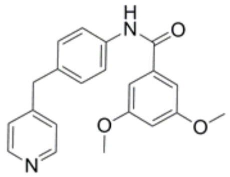
(5)	(4)	(4)

II-10 (3)

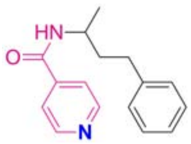	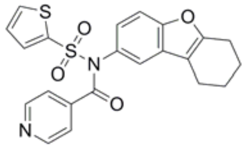	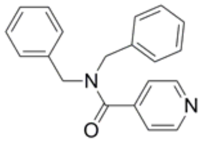
(5)	(4)	(4)

Cluster III

III-1 (5)

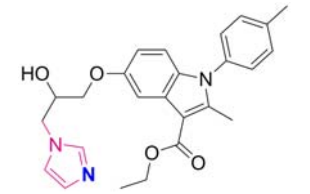	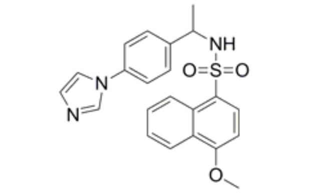	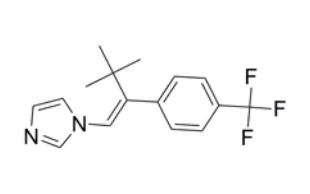	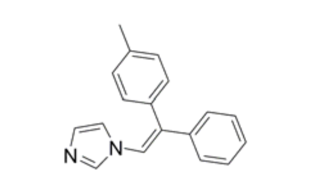
(4)	(4) EC_{50} 0.484 μM	(4) EC_{50} 0.754 μM	(4) EC_{50} 0.920 μM
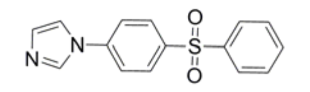			
(4)			

III-2 (7)

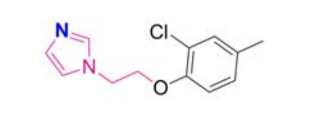	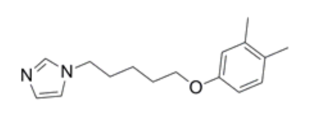	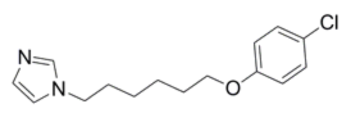	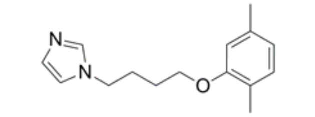
(5)	(5) EC_{50} 0.548 μM	(5) EC_{50} 0.948 μM	(4) EC_{50} 2.364 μM
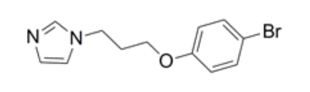	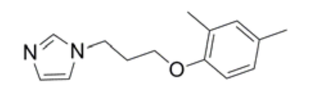	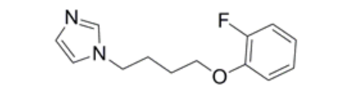	
(5) EC_{50} 3.664 μM	(5) EC_{50} 8.013 μM	(4)	

III-3 (4)

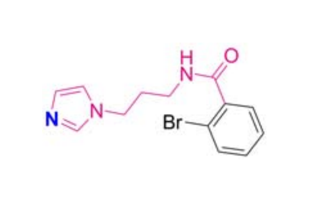	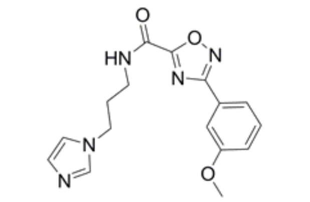	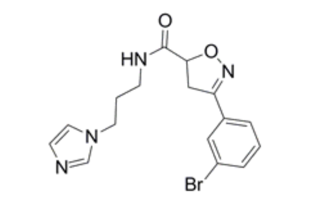	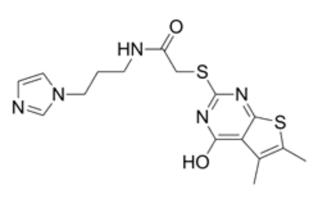
(4)	(4)	(4)	(4)

III-4 (3)

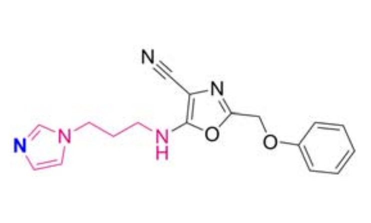	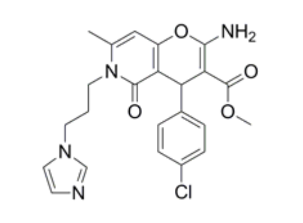	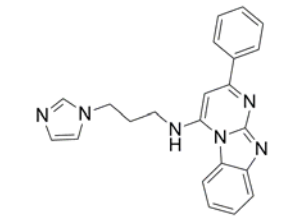
(4)	(4)	(4)

Cluster IV

IV-1 (1)

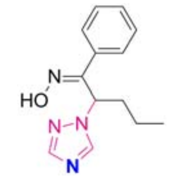
(4)

IV-2 (2)

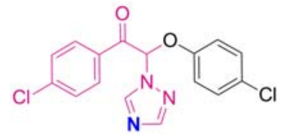	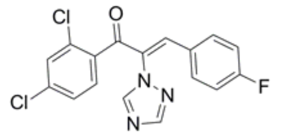
(5) EC_{50} 1.309 µM	(4) EC_{50} 7.804 µM

IV-3 (2)

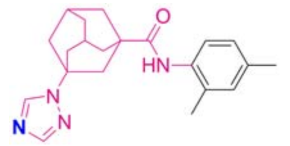	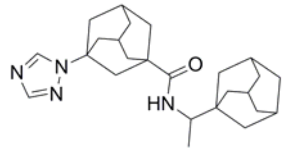
(5)	(4)

Cluster V

V-1 (1)

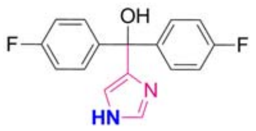

(4)

Cluster VI

VI-1 (1)

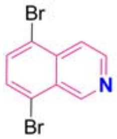

(4)
