# Supplementary material for: Diverse Inhibitor Chemotypes Targeting Trypanosoma cruzi CYP51
Source: PLoS Negl Trop Dis. 2012 Jul 31;6(7):e1736. doi: 10.1371/journal.pntd.0001736 (PMC3409115; doi:10.1371/journal.pntd.0001736)
Supplement: Table S1 — Analysis of plate readings. (DOCX) [file pntd.0001736.s002.docx]

**Table S1.** Analysis of plate readings

1. **Protein-plus test plates**

| Wavelength, nm | T=∑(Abs well A1 to P1) | U=∑(Abs well A2 to P2) | (T + U)/32 | Corrected absorbance in well A3 | Corrected absorbance in well A4 | Corrected absorbance in well A5 | etc… | Corrected absorbance in well A24 |
| --- | --- | --- | --- | --- | --- | --- | --- | --- |
| 350 | ∑(A1_350_+ B1_350_+…P1_350_) | ∑(A2_350_+ B2_350_+…P2_350_) | X_350_ | A3_350_-X_350_ | A4_350_-X_350_ | A5_350_-X_350_ | etc… | A24_350_-X_350_ |
| 360 | ∑(A1_360_+ B1_360_+…P1_360_) | ∑(A2_360_+ B2_360_+…P2_360_) | X_360_ | A3_360_-X_360_ | A4_360_-X_360_ | A5_360_-X_360_ | etc… | A24_360_-X_360_ |
| 370 | ∑(A1_370_+ B1_370_+…P1_370_) | ∑(A2_370_+ B2_370_+…P2_370_) | X_370_ | A3_370_-X_370_ | A4_370_-X_370_ | A5_370_-X_370_ | etc… | A24_370_-X_370_ |
| etc… | etc… | etc… | etc… | etc… | etc… | etc… | etc… | etc… |
| 500 | ∑(A1_500_+ B1_500_+…P1_500_) | ∑(A2_500_+ B2_500_+…P2_500_) | X_500_ | A3_500_-X_500_ | A4_500_-X_500_ | A5_500_-X_500_ | etc… | A24_500_-X_500_ |

1. **Protein-minus reference plates**

| Wavelength, nm | V=∑(Abs well A1 to P1) | W=∑(Abs well A2 to P2) | (V + W)/32 | Corrected absorbance in well A3 | Corrected absorbance in well A4 | Corrected absorbance in well A5 | etc… | Corrected absorbance in well A24 |
| --- | --- | --- | --- | --- | --- | --- | --- | --- |
| 350 | ∑(A1_350_+ B1_350_+…P1_350_) | ∑(A2_350_+ B2_350_+…P2_350_) | Y_350_ | A3_350_-Y_350_ | A4_350_-Y_350_ | A5_350_-Y_350_ | etc… | A24_350_-Y_350_ |
| 360 | ∑(A1_360_+ B1_360_+…P1_360_) | ∑(A2_360_+ B2_360_+…P2_360_) | Y_360_ | A3_360_-Y_360_ | A4_360_-Y_360_ | A5_360_-Y_360_ | etc… | A24_360_-Y_360_ |
| 370 | ∑(A1_370_+ B1_370_+…P1_370_) | ∑(A2_370_+ B2_370_+…P2_370_) | Y_370_ | A3_370_-Y_370_ | A4_370_-Y_370_ | A5_370_-Y_370_ | etc… | A24_370_-Y_370_ |
| etc… | etc… | etc… | etc… | etc… | etc… | etc… | etc… | etc… |
| 500 | ∑(A1_500_+ B1_500_+…P1_500_) | ∑(A2_500_+ B2_500_+…P2_500_) | Y_500_ | A3_500_-Y_500_ | A4_500_-Y_500_ | A5_500_-Y_500_ | etc… | A24_500_-Y_500_ |

Where A through P are absorbance values in corresponding wells in 384-well screening plates (**Fig. 1**) at specified wavelengths; T is the sum of absorbance values from A1 to P1 and U is the sum of absorbance values from A2 through P2 in plate **A** at specified wavelengths; V is the sum of absorbance values from A1 to P1 and W is the sum of absorbance values from A2 through P2 in plate **B** at specified wavelengths; X is an absorbance of the buffer, DMSO and protein background averaged across the columns 1 and 2 in plate **A** for each individual wavelength, Y is an absorbance of the buffer and DMSO background averaged across the columns 1 and 2 in plate **B** for each individual wavelength
