# Supplementary material for: Diverse Inhibitor Chemotypes Targeting Trypanosoma cruzi CYP51
Source: PLoS Negl Trop Dis. 2012 Jul 31;6(7):e1736. doi: 10.1371/journal.pntd.0001736 (PMC3409115; doi:10.1371/journal.pntd.0001736)
Supplement: Table S2 — First 22 hits validated positive both in two-wavelength and spectral screening modes. (DOCX) [file pntd.0001736.s003.docx]

**Table S2.** First 22 hits validated positive both in two-wavelength and spectral screening modes

| 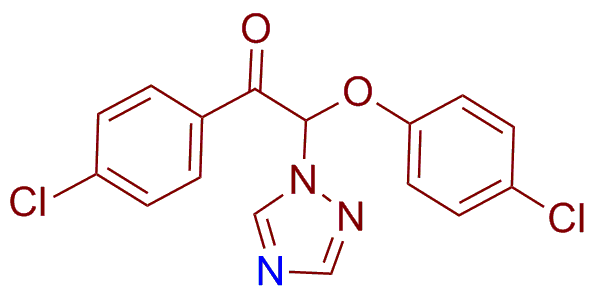 | 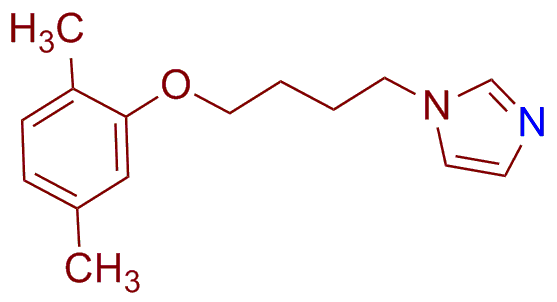 | 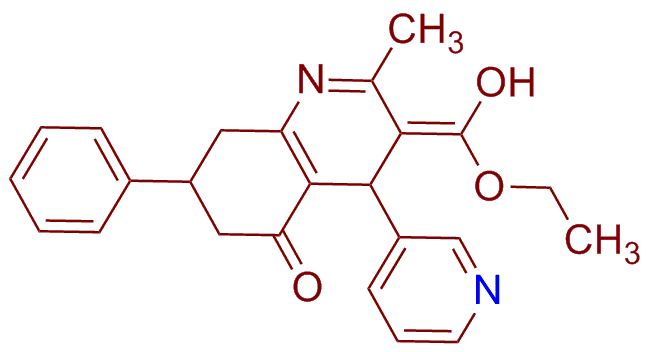 | 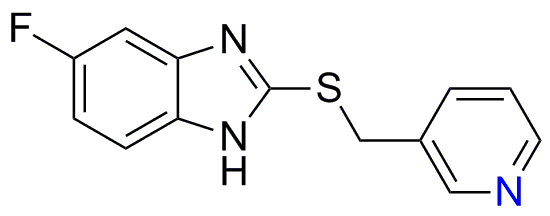 |
| --- | --- | --- | --- |
| (5)^1^ | (4) | (4) | (4) |
| 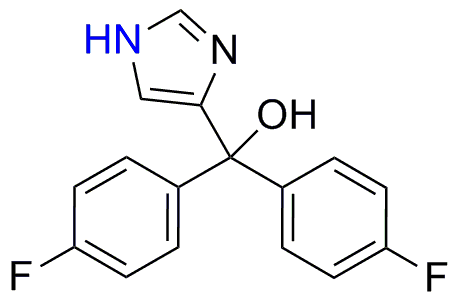 | 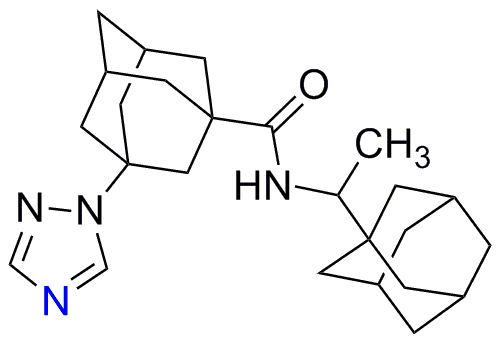 | 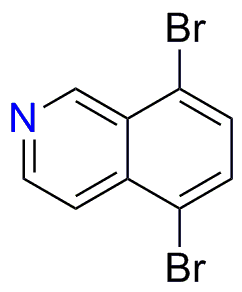 | 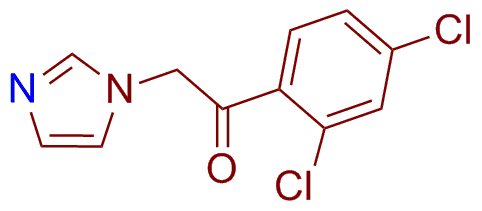 |
| (4) | (4) | (4) | (2) |
| 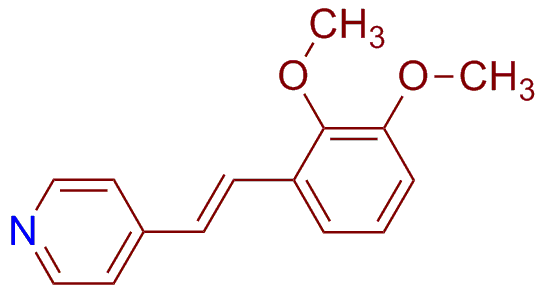 | 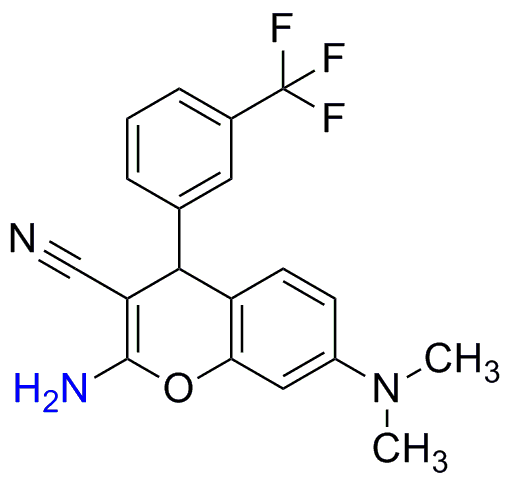 | 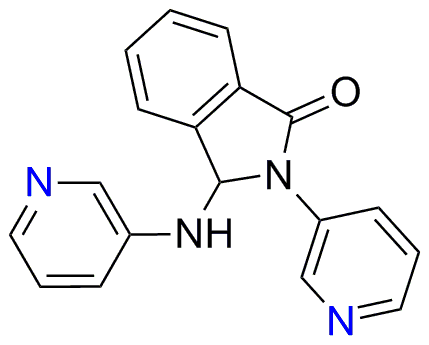 | 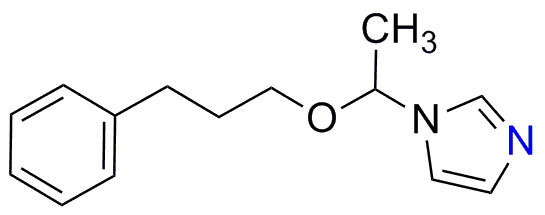 |
| (2) | (2) | (2) | (2) |
| 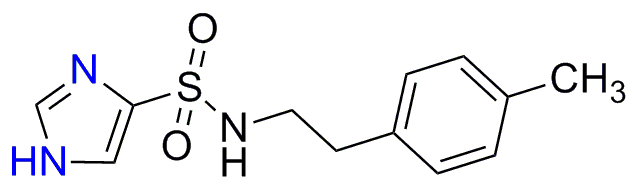 | 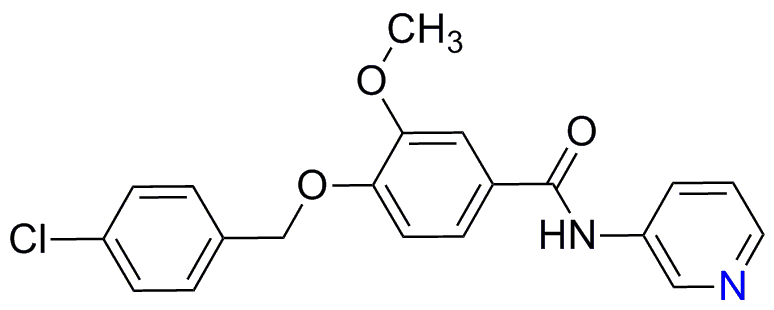 | 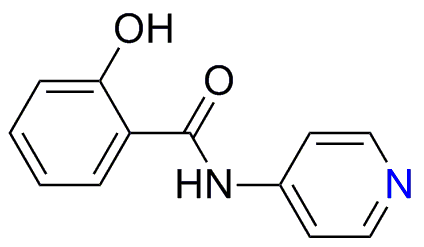 | 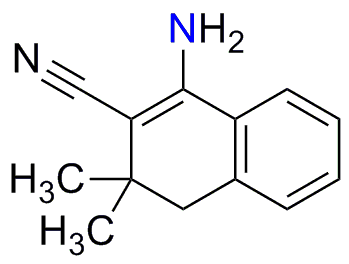 |
| (2) | (2) | (2) | (2) |
| 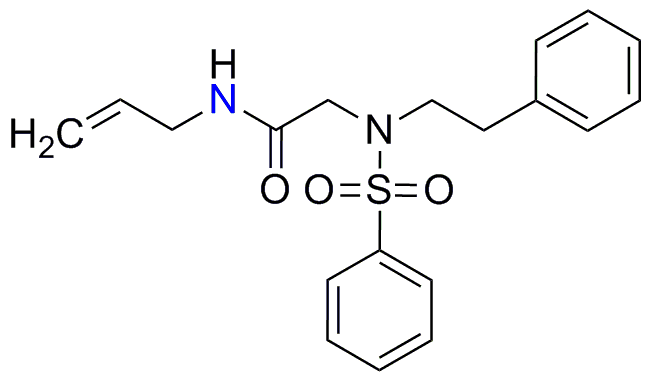 | 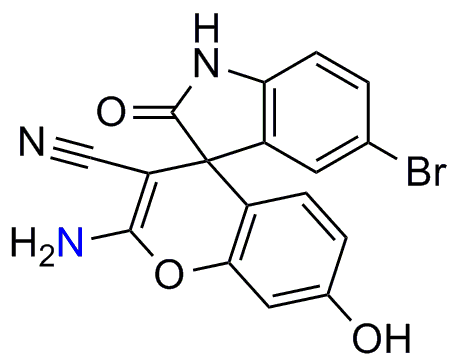 | 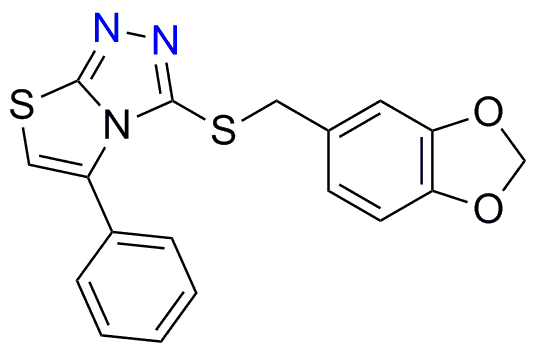 | 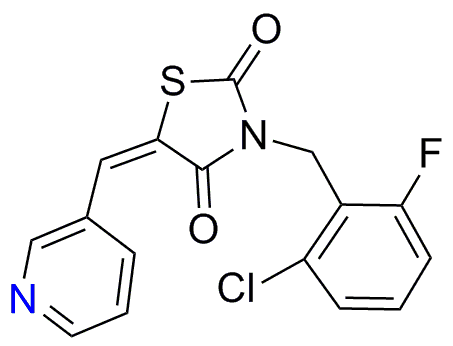 |
| (2) | (2) | (2) | (1) |
| 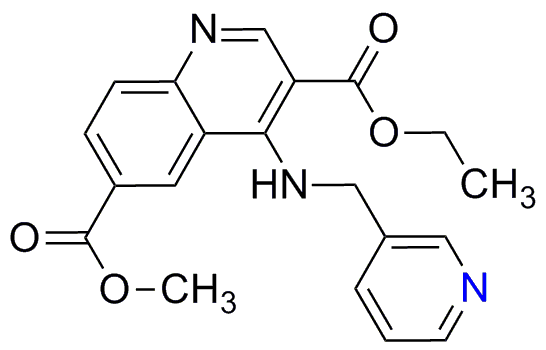 | 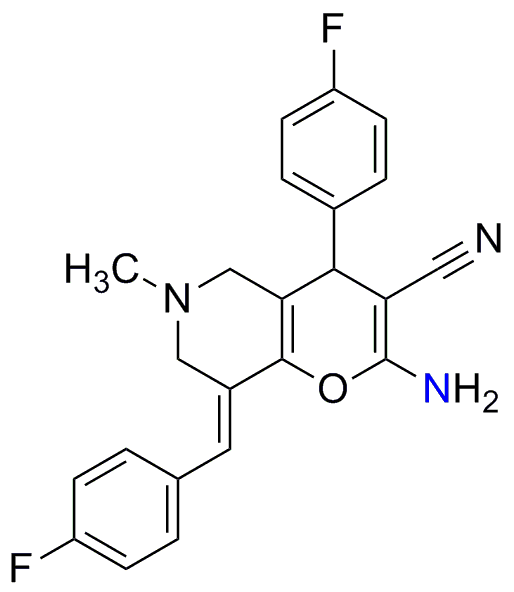 |  |  |
| (1) | (1) |  |  |

^1^Compound binding score is shown in parentheses.

17 hits possess nitrogen containing aromatic heterocycles. Nitrogen atom that likely coordinates to the heme iron is highlighted in blue. In red are shown compounds subsequently cross-validated active against *T. cruzi* cultured in mammalian cells.
