# Supplementary material for: Diverse Inhibitor Chemotypes Targeting Trypanosoma cruzi CYP51
Source: PLoS Negl Trop Dis. 2012 Jul 31;6(7):e1736. doi: 10.1371/journal.pntd.0001736 (PMC3409115; doi:10.1371/journal.pntd.0001736)
Supplement: Table S3 — 185 hits validated with binding score 4 or 5. (DOCX) [file pntd.0001736.s004.docx]

**Table S3.** 185 hits validated with binding score 4 or 5

|  | **Structure** | **Binding**  **score** | **Smiles^a^** | **MW** | **Calculated**  **logP** |
| --- | --- | --- | --- | --- | --- |
| 1 |  | 4 | CN(Cc1ccccc1)Cc2ccncc2 | 212.29 | 2.75 |
| 2 |  | 4 | O=C(CCC1CCCC1)Nc2ccncc2 | 218.30 | 2.48 |
| 3 |  | 4 | COC(=O)c1ccc(s1)c2ccncc2 | 219.26 | 2.64 |
| 4 |  | 5 | C(NC1Cc2ccccc2C1)c3cccnc3 | 224.30 | 3.17 |
| 5 |  | 4 | C1Oc2ccc(\C=C\c3ccncc3)cc2O1 | 225.24 | 2.63 |
| 6 |  | 5 | O=C(CCc1ccccc1)Nc2ccncc2 | 226.27 | 2.62 |
| 7 |  | 4 | CN(CCc1ccccc1)Cc2cccnc2 | 226.32 | 3.12 |
| 8 |  | 4 | CC(C)\C=C\1/CC\C(=C/c2ccncc2)\C1=O | 227.30 | 2.85 |
| 9 |  | 5 | Cc1ccc(OCCCn2ccnc2)c(C)c1 | 230.31 | 3.33 |
| 10 |  | 4 | Fc1ccccc1OCCCCn2ccnc2 | 234.27 | 2.95 |
| 11 |  | 5 | Cc1ccc(OCCn2ccnc2)c(Cl)c1 | 236.71 | 3.15 |
| 12 |  | 4 | CC(=O)NC(c1ccc(F)cc1)c2cccnc2 | 244.27 | 2.69 |
| 13 |  | 4 | CCCC(\C(=N/O)\c1ccccc1)n2cncn2 | 244.29 | 2.08 |
| 14 |  | 4 | Cc1ccc(C)c(OCCCCn2ccnc2)c1 | 244.33 | 3.70 |
| 15 |  | 4 | O=C(Nc1ccncc1)c2ccc3ccccc3c2 | 248.28 | 3.07 |
| 16 |  | 4 | N(c1cccnc1)c2nc(cs2)c3ccccc3 | 253.32 | 3.51 |
| 17 |  | 5 | O=C1C2C3CC(C=C3)C2C(=O)N1Cc4cccnc4 | 254.28 | 1.89 |
| 18 |  | 5 | CC(CCc1ccccc1)NC(=O)c2ccncc2 | 254.33 | 3.27 |
| 19 |  | 4 | CCC(Oc1ccccc1)C(=O)Nc2ccncc2 | 256.30 | 2.40 |
| 20 |  | 4 | C(C1CCC=CC1)N2CCCCC2c3cccnc3 | 256.39 | 3.34 |
| 21 |  | 5 | Cc1ccc(OCCCCCn2ccnc2)cc1C | 258.36 | 4.07 |
| 22 |  | 4 | Fc1ccc2[nH]c(SCc3cccnc3)nc2c1 | 259.30 | 3.01 |
| 23 |  | 4 | Cc1ccc(cc1)\C(=C/n2ccnc2)\c3ccccc3 | 260.33 | 4.18 |
| 24 |  | 4 | Clc1ccc(CC(=O)NCc2cccnc2)cc1 | 260.73 | 3.27 |
| 25 |  | 5 | Clc1ccc(NC(=O)NCc2cccnc2)cc1 | 261.72 | 2.55 |
| 26 |  | 4 | CCN1C(=O)CC(=Nc2ccccc12)c3ccncc3 | 265.31 | 2.60 |
| 27 |  | 4 | COc1ccccc1Cc2onc(n2)c3ccncc3 | 267.28 | 3.43 |
| 28 |  | 4 | Cc1cccc(Nc2nc(cs2)c3ccncc3)n1 | 268.34 | 3.28 |
| 29 |  | 4 | Oc1cccc(Nc2nc(cs2)c3ccncc3)c1 | 269.32 | 3.24 |
| 30 |  | 4 | CC(NCc1cccnc1)C23CC4CC(CC(C4)C2)C3 | 270.41 | 3.20 |
| 31 |  | 4 | Clc1ccc(Cc2onc(n2)c3ccncc3)cc1 | 271.71 | 4.12 |
| 32 |  | 5 | O=C(NCc1cccnc1)c2csc3CCCCc23 | 272.37 | 3.67 |
| 33 |  | 5 | Clc1ccc(OCCCCCCn2ccnc2)cc1 | 278.79 | 4.18 |
| 34 |  | 4 | Cc1ccc(CC2CC(=O)N(C2=O)c3ccncc3)cc1 | 280.32 | 2.70 |
| 35 |  | 4 | Cc1ccc(C)c(CN2CCCCC2c3cccnc3)c1 | 280.41 | 4.31 |
| 36 |  | 5 | Brc1ccc(OCCCn2ccnc2)cc1 | 281.15 | 3.11 |
| 37 |  | 4 | Clc1ccc(Cl)c(SCCc2ccncc2)c1 | 284.23 | 4.65 |
| 38 |  | 4 | COc1ccc(\C=C\C(=O)Nc2ccncc2)cc1OC | 284.31 | 2.25 |
| 39 |  | 4 | O=S(=O)(c1ccccc1)c2ccc(cc2)n3ccnc3 | 284.33 | 2.82 |
| 40 |  | 4 | CCC1=C(C#N)C(C(=C(N)O1)C(=O)OC)c2cccnc2 | 285.30 | 1.92 |
| 41 |  | 4 | CC(CCc1ccccc1)\N=C(/S)\Nc2cccnc2 | 285.41 | 3.70 |
| 42 |  | 4 | CC(C)N(Cc1ccccc1)\C(=N\c2cccnc2)\S | 285.41 | 3.38 |
| 43 |  | 4 | OC(c1c[nH]cn1)(c1ccc(F)cc1)c1ccc(F)cc1 | 286.28 | 3.46 |
| 44 |  | 4 | CCO\C(=C/1\C(c2cccnc2)n3nnnc3N=C1C)\O | 286.29 | 1.30 |
| 45 |  | 4 | Brc1ccc(Br)c2cnccc12 | 286.95 | 3.45 |
| 46 |  | 5 | C(NC(Cc1ccccc1)c2ccccc2)c3ccncc3 | 288.39 | 4.49 |
| 47 |  | 4 | Clc1ccc(CC(=O)ONC(=N)c2ccncc2)cc1 | 289.73 | 2.26 |
| 48 |  | 4 | CC1C(=Cccncc2)(=O)(=Cccncc3) | 290.36 | 3.20 |
| 49 |  | 4 | Cc1cc(Cl)ccc1OCCNC(=O)c2cccnc2 | 290.76 | 3.22 |
| 50 |  | 4 | CC(C)(C)\C(=C/n1ccnc1)\c2ccc(cc2)C(F)(F)F | 294.32 | 4.58 |
| 51 |  | 5 | CC(C)(C)c1ccc(\C=C\C(=O)NCc2cccnc2)cc1 | 294.39 | 4.20 |
| 52 |  | 4 | Brc1oc(cc1)C(=O)NCCc2ccncc2 | 295.13 | 2.430911 |
| 53 |  | 4 | Oc1ccccc1c2onc(c2)C(=O)NCc3cccnc3 | 295.29 | 2.63 |
| 54 |  | 4 | COc1ccccc1C(=O)n2nc(nc2N)c3cccnc3 | 295.30 | 1.72 |
| 55 |  | 4 | FC(F)(F)c1cccc(NC(=O)OCc2ccncc2)c1 | 296.25 | 3.51 |
| 56 |  | 4 | Cc1ccc(Cl)c(OP(=O)(C)Nc2cccnc2)c1 | 296.70 | 3.19 |
| 57 |  | 4 | Clc1ccccc1\C=C(/C#N)\C(=O)NCc2cccnc2 | 297.75 | 2.99 |
| 58 |  | 4 | CCC(C)(C)c1ccc(OCC(=O)Nc2ccncc2)cc1 | 298.38 | 3.61 |
| 59 |  | 5 | CCN(Cc1ccncc1)\C(=N\CCc2ccccc2)\S | 299.44 | 3.84 |
| 60 |  | 4 | Clc1ccccc1\C=C\C(=O)ONC(=N)c2ccncc2 | 301.74 | 2.35 |
| 61 |  | 4 | O=C(N(Cc1ccccc1)Cc2ccccc2)c3ccncc3 | 302.37 | 3.96 |
| 62 |  | 4 | Cc1cc(Cl)ccc1OCCCC(=O)Nc2cccnc2 | 304.78 | 3.60 |
| 63 |  | 5 | CCCCNC(=O)c1cc(nc2ccccc12)c3ccncc3 | 305.37 | 3.96 |
| 64 |  | 5 | CC(C)N(CCc1ccncc1)\C(=N\C2CCCCC2)\S | 305.48 | 3.77 |
| 65 |  | 4 | COc1ccc(cc1)c2[nH]nc3C(=O)NC(c4cccnc4)c23 | 306.32 | 2.07 |
| 66 |  | 4 | Brc1ccccc1C(=O)NCCCn2ccnc2 | 308.17 | 2.88 |
| 67 |  | 5 | CN1C(c2cccnc2)c3c(n[nH]c3C1=O)c4ccc(F)cc4 | 308.31 | 2.57 |
| 68 |  | 4 | COc1ccc(cc1)c2cc([nH]n2)C(=O)NCc3cccnc3 | 308.34 | 2.29 |
| 69 |  | 4 | Brc1oc(cc1)C(=O)ONC(=N)c2ccncc2 | 310.10 | 1.05 |
| 70 |  | 4 | Clc1ccc(NC(=O)NCCc2ccncc2)cc1Cl | 310.20 | 3.57 |
| 71 |  | 5 | Clc1ccc(cc1)S(=O)(=O)ONC(=N)c2ccncc2 | 311.76 | 1.77 |
| 72 |  | 4 | CCOc1cc(ccc1Cl)S(=O)(=O)Nc2cccnc2 | 312.78 | 2.51 |
| 73 |  | 4 | COC(=O)C1=C(N)OC2=C(C1c3cccnc3)C(=O)OC(=C2)C | 314.29 | 1.85 |
| 74 |  | 4 | CCCC\N=C(/S)\N1CCn2cccc2C1c3cccnc3 | 314.45 | 2.58 |
| 75 |  | 4 | O=C(Cc1coc2ccc3ccccc3c12)NCc4cccnc4 | 316.35 | 4.12 |
| 76 |  | 5 | O=C(N1CCCCC1)c2cc(nc3ccccc23)c4ccncc4 | 317.39 | 3.58 |
| 77 |  | 4 | Cc1cc(Cl)ccc1OCCCC(=O)NCc2cccnc2 | 318.81 | 3.97 |
| 78 |  | 4 | Cc1cc(Cl)ccc1OCCCC(=O)NCc2ccncc2 | 318.81 | 3.97 |
| 79 |  | 4 | CCn1c(cc2oc3ccccc3c12)C(=O)NCc4cccnc4 | 319.36 | 3.39 |
| 80 |  | 4 | Oc1cccc(CC(=O)Nc2cc(cnc2O)c3ccncc3)c1 | 321.33 | 2.76 |
| 81 |  | 4 | N#Cc1nc(COc2ccccc2)oc1NCCCn3ccnc3 | 323.35 | 2.67 |
| 82 |  | 4 | FC(F)(F)Oc1ccc(NC(=O)CNCc2cccnc2)cc1 | 325.29 | 2.44 |
| 83 |  | 4 | CC1=NN(C(=O)COc2ccccc2C)C(O)(C1)c3cccnc3 | 325.36 | 1.87 |
| 84 |  | 4 | COc1cccc(c1)c2noc(n2)C(=O)NCCCn3ccnc3 | 327.34 | 2.63 |
| 85 |  | 4 | Cc1cc(C=C2C(=O)c3ccccc3C2=O)c(C)n1c4ccncc4 | 328.36 | 3.47 |
| 86 |  | 4 | Clc1ccc(cc1)c2noc(CCC(=O)Nc3cccnc3)n2 | 328.76 | 3.76 |
| 87 |  | 4 | Cc1[nH]c2ccccc2c1C(Nc3ccc(F)cc3)c4ccncc4 | 331.39 | 4.99 |
| 88 |  | 4 | Cc1[nH]c2ccccc2c1C(Nc3cccc(F)c3)c4cccnc4 | 331.39 | 4.99 |
| 89 |  | 4 | COc1ccccc1C(=O)O\N=C(/c2ccccc2)\c3ccncc3 | 332.35 | 3.63 |
| 90 |  | 5 | COc1ccc2nc3oc(cc3cc2c1)C(=O)NCc4cccnc4 | 333.34 | 3.16 |
| 91 |  | 5 | COc1ccc(NC(=O)Nc2ccc(Cc3ccncc3)cc2)cc1 | 333.39 | 3.60 |
| 92 |  | 4 | Cc1c(sc2nc(cn12)c3ccccc3)C(=O)Nc4ccncc4 | 334.40 | 3.10 |
| 93 |  | 4 | O=C(NCc1ccncc1)C(Sc2ccccc2)c3ccccc3 | 334.44 | 4.20 |
| 94 |  | 4 | OC1=C(C(=O)NCc2ccncc2)C(=O)N(CC=C)c3ccccc13 | 335.36 | 2.18 |
| 95 |  | 5 | O=C(NCc1cccnc1)c2cc3C(=O)Oc4ccccc4c3s2 | 336.37 | 3.74 |
| 96 |  | 4 | COC(=O)c1sccc1NC(=O)CSCCc2ccncc2 | 336.43 | 3.04 |
| 97 |  | 5 | CCc1nc(SCc2cccnc2)c(C#N)c(c1C)C(F)(F)F | 337.36 | 4.62 |
| 98 |  | 5 | Clc1ccc(cc1)n2ncc3C(=O)N(Cc4cccnc4)C=Nc23 | 337.77 | 3.16 |
| 99 |  | 4 | CC1=Nc2ncnn2C(/C/1=C()/OC3CCCCC3)c4cccnc4 | 339.39 | 2.31 |
| 100 |  | 4 | Nc1cc(NC(=O)c2cccc(Cl)c2)cc(Oc3cccnc3)c1 | 339.79 | 3.31 |
| 101 |  | 4 | NC1=C(C#N)C(c2ccncc2)c3ccc(Nc4ccccc4)cc3O1 | 340.38 | 3.72 |
| 102 |  | 4 | Clc1cccc(Cl)c1CSCC(=O)NCc2cccnc2 | 341.28 | 4.30 |
| 103 |  | 4 | Clc1ccccc1OCc2oc(cc2)C(=O)NCc3cccnc3 | 342.79 | 3.62 |
| 104 |  | 5 | CCOC(=O)C1=C(C)N(C)C(=C(C1c2ccncc2)C(=O)OCC)C | 344.41 | 3.40 |
| 105 |  | 4 | CC(C)(C)Nc1c(nc2ccc(Br)cn12)c3ccncc3 | 345.24 | 3.65 |
| 106 |  | 4 | COc1ccc(cc1)C(=O)C(OC(=O)c2ccncc2)c3ccccc3 | 347.37 | 3.35 |
| 107 |  | 5 | Clc1ccc(OC(C(=O)c2ccc(Cl)cc2)n2cncn2)cc1 | 348.20 | 3.63 |
| 108 |  | 4 | COc1cc(OC)cc(c1)C(=O)Nc2ccc(Cc3ccncc3)cc2 | 348.40 | 3.90 |
| 109 |  | 5 | Cc1nnc(NCc2cccnc2)c3c1nnn3Cc4ccc(F)cc4 | 349.37 | 3.22 |
| 110 |  | 5 | Cc1ccc(cc1)S(=O)(=O)NCCCOC(=O)Nc2ccncc2 | 349.41 | 2.08 |
| 111 |  | 5 | Cc1ccc(NC(=O)C23CC4CC(C2)CC(C4)(C3)n5cncn5)c(C)c1 | 350.46 | 3.58 |
| 112 |  | 4 | Cc1ccc(Cl)cc1n2ncc3c(NCc4cccnc4)ncnc23 | 350.82 | 4.23 |
| 113 |  | 4 | Cc1ccccc1CSCc2oc(cc2)C(=O)NCc3cccnc3 | 352.45 | 4.31 |
| 114 |  | 5 | CC1(C)CC(=O)C2=C(C1)c3c(NC2c4cccnc4)ccc5ccccc35 | 354.45 | 4.83 |
| 115 |  | 5 | C\C(=N/OC(=O)c1c(C)onc1c2ccccc2Cl)\c3ccncc3 | 355.79 | 4.15 |
| 116 |  | 4 | Fc1ccc(\C=C(\C(=O)c2ccc(Cl)cc2Cl)/n3cncn3)cc1 | 362.21 | 4.27 |
| 117 |  | 4 | C(Cn1nnc(n1)c2ccncc2)OCCn3nnc(n3)c4ccncc4 | 364.37 | 2.73 |
| 118 |  | 4 | CCc1cc2c(cc3C(=O)N(CC(=O)NCc4cccnc4)N=Cn23)s1 | 367.43 | 2.33 |
| 119 |  | 4 | C(CNc1cc(nc2nc3ccccc3n12)c4ccccc4)Cn5ccnc5 | 368.44 | 4.40 |
| 120 |  | 4 | COc1cccc(CNCc2cccnc2)c1OCc3ccc(Cl)cc3 | 368.87 | 4.77 |
| 121 |  | 4 | O=S(=O)(N1CCCC1)c2ccc(cc2)c3csc(n3)c4cccnc4 | 371.48 | 3.61 |
| 122 |  | 5 | COc1ccc(cc1)c2nc3N=C4CCCC(=C4C(c5cccnc5)n3n2)O | 373.41 | 3.87 |
| 123 |  | 4 | Cc1ccc(Sc2c([nH]c3ccccc23)C(=O)NCc4cccnc4)cc1 | 373.47 | 4.63 |
| 124 |  | 4 | COCc1cc(C)nc2N(Cc3ccccc3)C(NC(=O)c12)c4cccnc4 | 374.44 | 4.05 |
| 125 |  | 4 | Fc1ccc(NC(=O)c2ccc3C(=O)N(Cc4cccnc4)C(=O)c3c2)cc1 | 375.35 | 3.00 |
| 126 |  | 4 | Brc1cccc(c1)C2=NOC(C2)C(=O)NCCCn3ccnc3 | 377.24 | 3.41 |
| 127 |  | 4 | Cc1sc2nc(SCC(=O)NCCCn3ccnc3)nc(O)c2c1C | 377.49 | 3.61 |
| 128 |  | 4 | OC1=C2C(c3ccc(Cl)cc3)n4nc(nc4N=C2CCC1)c5cccnc5 | 377.84 | 4.56 |
| 129 |  | 4 | CC1=C(C(C2=C(O)CC(C)(C)CC2=N1)c3ccncc3)C(=O)OC4CCCC4 | 380.48 | 4.39 |
| 130 |  | 4 | O=C1C2(CN3CC1(CN(C2)C3c4cccnc4)c5ccccc5)c6ccccc6 | 381.47 | 3.23 |
| 131 |  | 4 | O=C1CN(C2CCCCC2)C(=O)C(N1CCC3=CCCCC3)c4ccncc4 | 381.51 | 3.57 |
| 132 |  | 5 | COc1ccc(CNc2nc3ccccc3c4nc(nn24)c5ccncc5)cc1 | 382.42 | 4.63 |
| 133 |  | 4 | COc1ccc(cc1)c2oc(C)c(CSCC(=O)NCc3cccnc3)n2 | 383.47 | 3.72 |
| 134 |  | 5 | Cn1c(SCc2onc(n2)c3ccc(Cl)cc3)nnc1c4ccncc4 | 384.85 | 4.58 |
| 135 |  | 4 | Clc1cccc(c1)S(=O)(=O)Nc2ccc(cc2)C(=O)Nc3ccncc3 | 387.85 | 3.20 |
| 136 |  | 4 | CCO\C(O)=C1C(c2cccnc2)C2=C(CC(CC2=O)c2ccccc2)N=C/1C | 388.46 | 3.58 |
| 137 |  | 4 | COc1ccc(cc1OC)c2cc(nc(NCc3cccnc3)n2)C(F)(F)F | 390.36 | 4.40 |
| 138 |  | 5 | Clc1ccccc1OP(=O)(Nc2cccnc2)Oc3ccccc3Cl | 395.20 | 4.16 |
| 139 |  | 5 | O=C(CN(Cc1ccccc1)S(=O)(=O)c2ccccc2)NCc3ccncc3 | 395.48 | 2.96 |
| 140 |  | 5 | Cc1n[nH]c(C)c1CCc2nc3c4ccccc4nc(NCc5cccnc5)n3n2 | 398.47 | 4.67 |
| 141 |  | 4 | O=C(CCCCCN1C(=O)c2cccc3cccc(C1=O)c23)NCc4cccnc4 | 401.46 | 3.99 |
| 142 |  | 4 | Brc1ccc(cc1)S(=O)(=O)Nc2ccc(Cc3ccncc3)cc2 | 403.29 | 4.35 |
| 143 |  | 5 | FC(F)(Cl)c1cc(nc2cc(nn12)C(=O)NCc3cccnc3)c4occc4 | 403.78 | 3.40 |
| 144 |  | 4 | CC(C)c1nnc(s1)N2C(C3=C(Oc4ccccc4C3=O)C2=O)c5cccnc5 | 404.44 | 3.30 |
| 145 |  | 4 | Clc1ccc(cc1)C2=NN(CC(=O)NCc3cccnc3)C(=O)c4ccccc24 | 404.86 | 3.92 |
| 146 |  | 5 | CCOC(=O)c1cc(c2ccc(C)cc2)n(CCC(=O)NCc3cccnc3)c1C | 405.49 | 4.79 |
| 147 |  | 4 | COc1ccc(c2ccccc12)S(=O)(=O)NC(C)c3ccc(cc3)n4ccnc4 | 407.49 | 3.83 |
| 148 |  | 4 | CC(NC(=O)C12CC3CC(C1)CC(C3)(C2)n1cncn1)C12CC3CC(CC(C3)C1)C2 | 408.58 | 3.69 |
| 149 |  | 5 | Brc1ccc(cc1)S(=O)(=O)NCc2csc(n2)c3cccnc3 | 410.31 | 3.42 |
| 150 |  | 4 | FC(F)(F)c1cc(nc(SCC(=O)NCc2ccncc2)n1)c3cccs3 | 410.44 | 4.57 |
| 151 |  | 5 | OC(C(C(=O)c1ccc(Cl)cc1Cl)c2cccnc2)C(Cl)(Cl)Cl | 413.56 | 4.51 |
| 152 |  | 4 | Cc1ccc2nc(NC(=O)C3CCN(CC3)S(=O)(=O)c4cccnc4)sc2c1 | 416.52 | 2.74 |
| 153 |  | 4 | Cc1coc(NC(=O)c2sc3nc(cc(c3c2N)C(F)(F)F)c4ccncc4)n1 | 419.38 | 4.01 |
| 154 |  | 4 | COc1ccc(CNc2nc(nn2S(=O)(=O)c3ccccc3)c4cccnc4)cc1 | 421.47 | 3.60 |
| 155 |  | 4 | Fc1ccc(cc1)c2cc(nc(SCC(=O)NCc3ccncc3)n2)C(F)(F)F | 422.40 | 4.65 |
| 156 |  | 4 | FC(F)(F)c1cc(nc(SCCCC(=O)NCc2ccncc2)n1)c3occc3 | 422.43 | 4.34 |
| 157 |  | 4 | Cc1c(sc2N=C3CCCCCN3C(=O)c12)C(=O)N4CCCCC4c5cccnc5 | 422.55 | 3.93 |
| 158 |  | 4 | NC1=C(C#N)C(c2cccnc2)c3c(N)nc(SCc4ccc(Cl)cc4)nc3O1 | 422.90 | 3.79 |
| 159 |  | 4 | CC1=C(C(C2=C(O)CC(C)(C)CC2=N1)c3cccnc3)C(=O)Nc4ccc(F)cc4F | 423.46 | 4.62 |
| 160 |  | 4 | CC1=C(C(c2ccc(Cl)cc2)n3nc(CCCO)nc3N1)C(=O)Nc4cccnc4 | 424.89 | 3.88 |
| 161 |  | 4 | CN1C(=O)N(C)c2nc(NCc3cccnc3)n(Cc4cccc5ccccc45)c2C1=O | 426.47 | 4.01 |
| 162 |  | 4 | COc1cc(OC)cc(c1)c2nc3N=C4CC(C)(C)CC(=C4C(c5ccncc5)n3n2)O | 431.49 | 4.28 |
| 163 |  | 4 | COc1cc(OC)cc(c1)c2cc3nc(C)c(CCC(=O)Nc4cccnc4)c(C)n3n2 | 431.49 | 4.23 |
| 164 |  | 4 | CC1Cc2cc(ccc2O1)C(=O)C3=C(O)C(=O)N(Cc4cccnc4)C3c5cccs5 | 432.49 | 3.91 |
| 165 |  | 4 | CCOC(=O)c1c(C)n(c2ccc(C)cc2)c3ccc(OCC(O)Cn4ccnc4)cc13 | 433.50 | 4.55 |
| 166 |  | 5 | COc1ccc(NC(=O)C2=C(C)Nc3nc(nn3C2c4ccncc4)c5ccccc5)cc1 | 438.48 | 4.50 |
| 167 |  | 4 | O=C(N(c1ccc2oc3CCCCc3c2c1)S(=O)(=O)c4cccs4)c5ccncc5 | 438.52 | 4.22 |
| 168 |  | 4 | Cc1sc(NC(=O)c2occc2)c(C(N3CCN(CCO)CC3)c4cccnc4)c1C | 440.56 | 3.26 |
| 169 |  | 4 | COc1cccc(c1)C2C(=C(C)Nc3nc(nn23)c4cccs4)C(=O)Nc5cccnc5 | 444.51 | 4.58 |
| 170 |  | 4 | COc1ccc(cc1)c2nc3NC(=C(C(c4cccs4)n3n2)C(=O)Nc5cccnc5)C | 444.51 | 4.58 |
| 171 |  | 5 | COc1cc(OC)cc(c1)c2cc3nc(C)c(CCC(=O)NCc4cccnc4)c(C)n3n2 | 445.52 | 4.60 |
| 172 |  | 4 | Cc1ccc(cc1)S(=O)(=O)N(CC(=O)Nc2cccnc2)c3cccc(c3)C(F)(F)F | 449.45 | 4.09 |
| 173 |  | 4 | Clc1ccc(N(CC(=O)NCc2ccncc2)S(=O)(=O)c3ccccc3)c(Cl)c1 | 450.36 | 4.19 |
| 174 |  | 4 | COc1ccc(cc1OC)c2noc(CSc3nnc(c4ccncc4)n3CC(C)C)n2 | 452.53 | 4.87 |
| 175 |  | 4 | COC(=O)C1=C(N)OC2=C(C1c3ccc(Cl)cc3)C(=O)N(CCCn4ccnc4)C(=C2)C | 454.92 | 3.87 |
| 176 |  | 4 | CC(C)(C)NC(=O)c1cccc(NC(=O)c2ccc3C(=O)N(Cc4cccnc4)C(=O)c3c2)c1 | 456.49 | 3.60 |
| 177 |  | 4 | COc1cc(NS(=O)(=O)c2ccc(Nc3nc(cs3)c4cccnc4)cc2)nc(OC)n1 | 470.53 | 3.11 |
| 178 |  | 4 | O=C(NCc1cccnc1)\C(=C/c2cccc(Oc3ccccc3)c2)\S(=O)(=O)c4ccccc4 | 470.54 | 4.69 |
| 179 |  | 4 | CC1=C(C(c2cccc(O)c2)n3nc(SCc4ccccc4)nc3N1)C(=O)Nc5cccnc5 | 470.55 | 4.65 |
| 180 |  | 4 | Cc1ccc(cc1)S(=O)(=O)N(CC(=O)NCc2cccnc2)c3cccc(Br)c3 | 474.37 | 4.04 |
| 181 |  | 5 | CCOc1cc(ccc1OCC=C)C2N(Cc3ccncc3)C(=O)c4[nH]nc(c5ccccc5O)c24 | 482.53 | 4.70 |
| 182 |  | 4 | COc1cccc(C2C(=C(C)Nc3nc(nn23)c4cccc(C)c4)C(=O)Nc5cccnc5)c1OC | 482.54 | 4.91 |
| 183 |  | 4 | CCCCOc1ccc(cc1OC)C2N(Cc3cccnc3)C(=O)c4[nH]nc(c5ccccc5O)c24 | 484.55 | 4.83 |
| 184 |  | 5 | CC1=C(C(C(=C(N1)SCC(=O)Nc2ccc(F)cc2)C#N)c3cccnc3)C(=O)Nc4ccccc4 | 499.56 | 4.48 |
| 185 |  | 4 | COc1cc2C(C(N(C)C(=O)c2cc1OC)c3cccnc3)C(=O)N4CCC(Cc5ccccc5)CC4 | 499.60 | 4.58 |
