# Supplementary material for: Diverse Inhibitor Chemotypes Targeting Trypanosoma cruzi CYP51
Source: PLoS Negl Trop Dis. 2012 Jul 31;6(7):e1736. doi: 10.1371/journal.pntd.0001736 (PMC3409115; doi:10.1371/journal.pntd.0001736)
Supplement: Table S4 — 57 T. cruzi -active hits in descending rank order. (DOCX) [file pntd.0001736.s005.docx]

**Table S4.** 57 *T. cruzi*-active hits in descending rank order

| Rank  Order | Structure | Binding score | EC_50_ (μM) | Toxicity  score^a^ | Smiles | MW | logP |
| --- | --- | --- | --- | --- | --- | --- | --- |
| 1 | 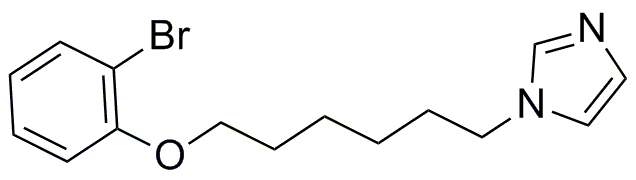 | 2 | 0.070 | 2 | Brc1ccccc1OCCCCCCn2ccnc2 | 323.23 | 4.23 |
| 2 | 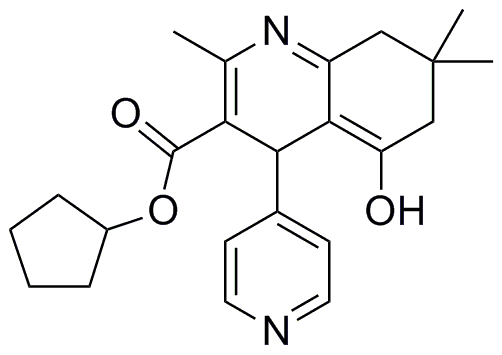 | 4 | 0.306 | 4 | CC1=C(C(C2=C(O)CC(C)(C)CC2=N1)c3ccncc3)C(=O)OC4CCCC4 | 380.48 | 4.39 |
| 3 | 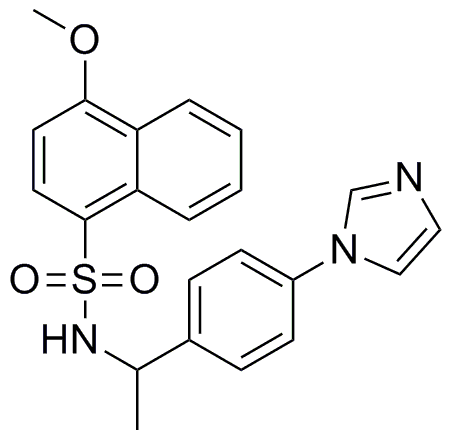 | 4 | 0.484 | 2 | COc1ccc(c2ccccc12)S(=O)(=O)NC(C)c3ccc(cc3)n4ccnc4 | 407.49 | 3.83 |
| 4 | 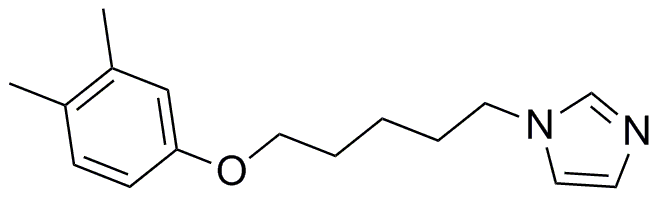 | 5 | 0.548 | 3 | Cc1ccc(OCCCCCn2ccnc2)cc1C | 258.36 | 4.07 |
| 5 | 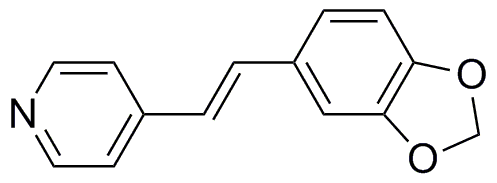 | 4 | 0.564 | 1 | C1Oc2ccc(\C=C\c3ccncc3)cc2O1 | 225.24 | 2.63 |
| 6 | 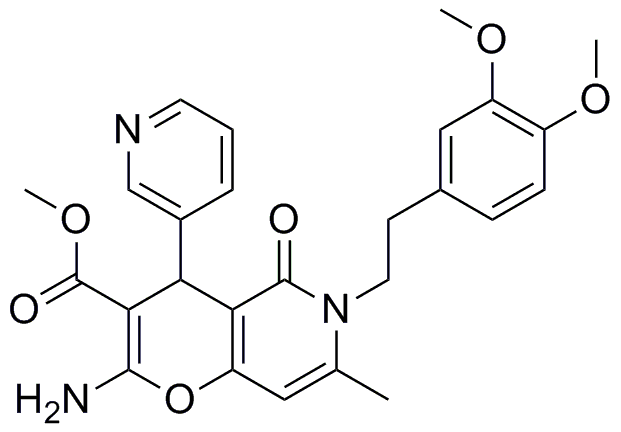 | 3 | 0.614 | 1 | COC(=O)C1=C(N)OC2=C(C1c3cccnc3)C(=O)N(CCc4ccc(OC)c(OC)c4)C(=C2)C | 477.51 | 3.57 |
| 7 | 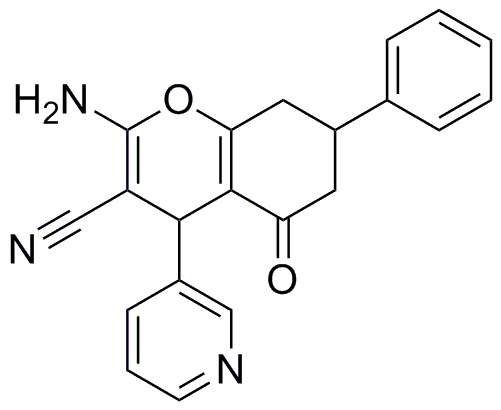 | 3 | 0.667 | 4 | NC1=C(C#N)C(C2=C(CC(CC2=O)c3ccccc3)O1)c4cccnc4 | 343.38 | 3.133 |
| 8 | 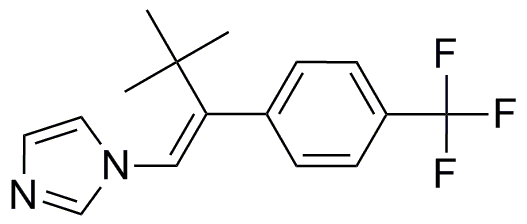 | 4 | 0.754 | 3 | CC(C)(C)\C(=C/n1ccnc1)\c2ccc(cc2)C(F)(F)F | 294.32 | 4.58 |
| 9 | 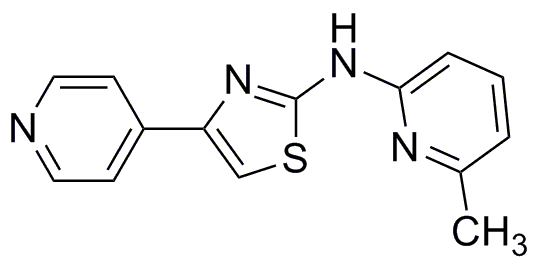 | 4 | 0.914 | 1 | Cc1cccc(Nc2nc(cs2)c3ccncc3)n1 | 268.34 | 3.28 |
| 10 | 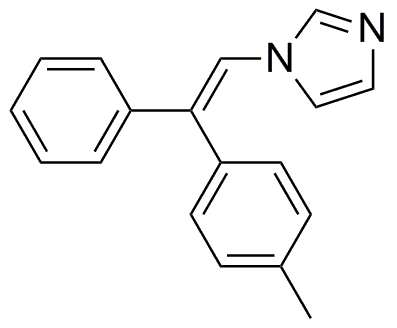 | 4 | 0.920 | 4 | Cc1ccc(cc1)\C(=C/n2ccnc2)\c3ccccc3 | 260.33 | 4.18 |
| 11 | 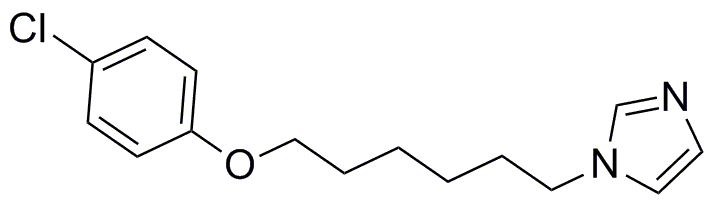 | 5 | 0.948 | 2 | Clc1ccc(OCCCCCCn2ccnc2)cc1 | 278.79 | 4.18 |
| 12 | 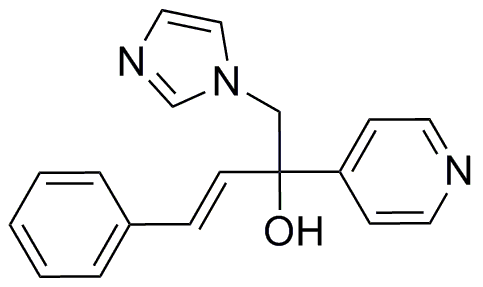 | 1 | 1.100 | 1 | OC(Cn1ccnc1)(\C=C\c2ccccc2)c3ccncc3 | 291.35 | 3.12 |
| 13 | 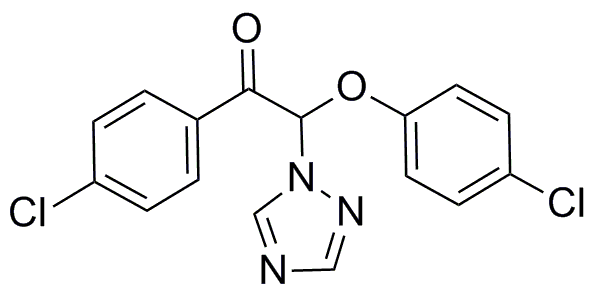 | 5 | 1.309 | 3 | Clc1ccc(OC(C(=O)c2ccc(Cl)cc2)n2cncn2)cc1 | 348.20 | 3.63 |
| 14 | 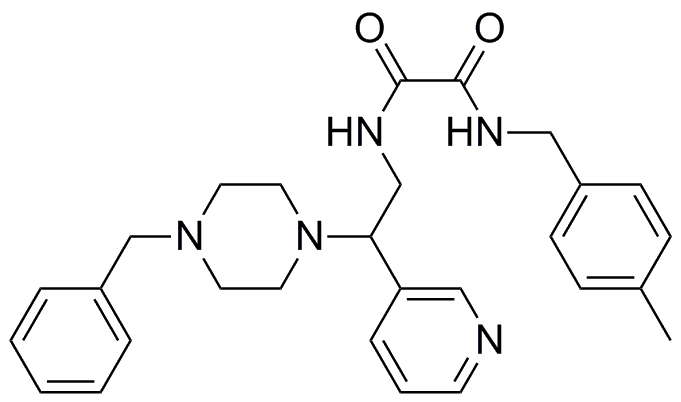 | 2 | 1.671 | 4 | Cc1ccc(CNC(=O)C(=O)NCC(N2CCN(Cc3ccccc3)CC2)c4cccnc4)cc1 | 471.60 | 3.94 |
| 15 | 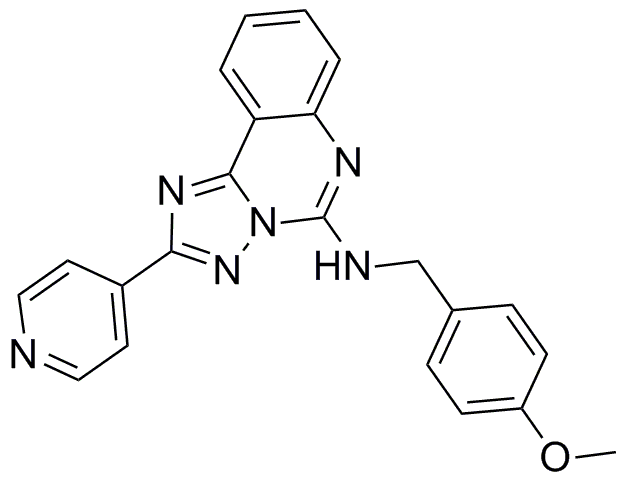 | 5 | 1.929 | 3 | COc1ccc(CNc2nc3ccccc3c4nc(nn24)c5ccncc5)cc1 | 382.42 | 4.63 |
| 16 | 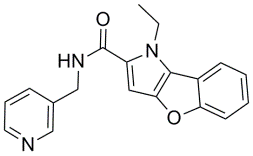 | 4 | 2.200 | 4 | CCn1c(cc2oc3ccccc3c12)C(=O)NCc4cccnc4 | 319.36 | 3.39 |
| 17 | 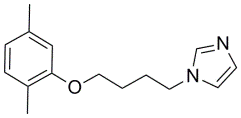 | 4 | 2.364 | N/D | Cc1ccc(C)c(OCCCCn2ccnc2)c1 | 244.33 | 3.70 |
| 18 | 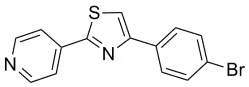 | 3 | 2.377 | 1 | Brc1ccc(cc1)c2csc(n2)c3ccncc3 | 317.21 | 4.56 |
| 19 | 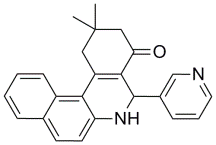 | 5 | 2.497 | 3 | CC1(C)CC(=O)C2=C(C1)c3c(NC2c4cccnc4)ccc5ccccc35 | 354.45 | 4.83 |
| 20 | 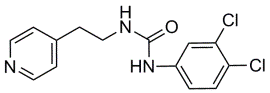 | 4 | 2.548 | 1 | Clc1ccc(NC(=O)NCCc2ccncc2)cc1Cl | 310.20 | 3.57 |
| 21 | 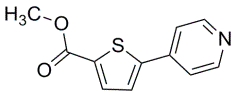 | 4 | 2.738 | 2 | COC(=O)c1ccc(s1)c2ccncc2 | 219.26 | 2.64 |
| 22 | 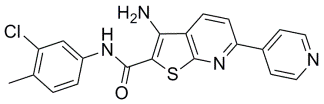 | 2 | 2.863 | 2 | Cc1ccc(NC(=O)c2sc3nc(ccc3c2N)c4ccncc4)cc1Cl | 394.89 | 4.98 |
| 23 | 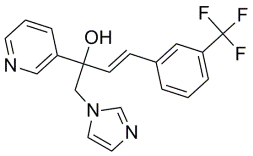 | 1 | 2.890 | 1 | OC(Cn1ccnc1)(\C=C\c2cccc(c2)C(F)(F)F)c3cccnc3 | 359.35 | 4.23 |
| 24 | 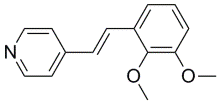 | 2 | 2.975 | N/D | COc1cccc(\C=C\c2ccncc2)c1OC | 241.29 | 2.98 |
| 25 | 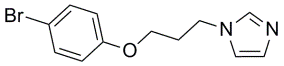 | 5 | 3.664 | 2 | Brc1ccc(OCCCn2ccnc2)cc1 | 281.15 | 3.11 |
| 26 | 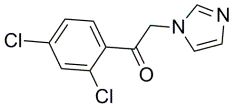 | 2 | 4.532 | N/D | Clc1ccc(C(=O)Cn2ccnc2)c(Cl)c1 | 255.12 | 2.81 |
| 27 | 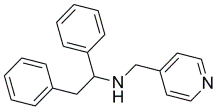 | 5 | 4.822 | 1 | C(NC(Cc1ccccc1)c2ccccc2)c3ccncc3 | 288.39 | 4.49 |
| 28 | 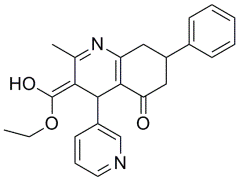 | 4 | 5.0 | N/D | CCO\C(O)=C1C(c2cccnc2)C2=C(CC(CC2=O)c2ccccc2)N=C/1C | 388.46 | 3.58 |
| 29 | 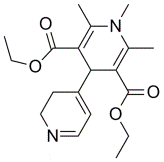 | 5 | 5.179 | 1 | CCOC(=O)C1=C(C)N(C)C(=C(C1c2ccncc2)C(=O)OCC)C | 344.41 | 3.40 |
| 30 | 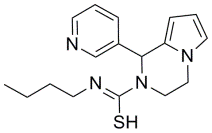 | 4 | 6.335 | 1 | CCCC\N=C(/S)\N1CCn2cccc2C1c3cccnc3 | 314.45 | 2.58 |
| 31 | 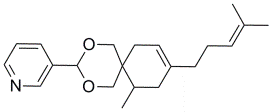 | 2 | 6.393 | 1 | CC1CC(=CCC12COC(OC2)c3cccnc3)CCC=C(C)C | 327.46 | 4.22 |
| 32 | 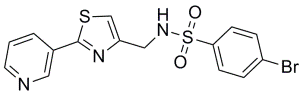 | 5 | 6.504 | 2 | Brc1ccc(cc1)S(=O)(=O)NCc2csc(n2)c3cccnc3 | 410.31 | 3.42 |
| 33 | 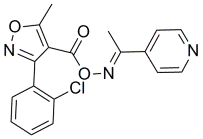 | 5 | 6.779 | 3 | C\C(=N/OC(=O)c1c(C)onc1c2ccccc2Cl)\c3ccncc3 | 355.79 | 4.15 |
| 34 | 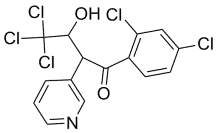 | 5 | 6.873 | 2 | OC(C(C(=O)c1ccc(Cl)cc1Cl)c2cccnc2)C(Cl)(Cl)Cl | 413.56 | 4.51 |
| 35 | 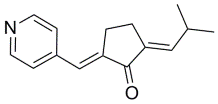 | 4 | 7.148 | 1 | CC(C)\C=C\1/CC\C(=C/c2ccncc2)\C1=O | 227.30 | 2.85 |
| 36 | 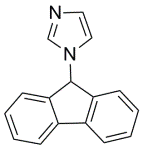 | 1 | 7.236 | 3 | c1ccc2c(c1)C(c3ccccc23)n4ccnc4 | 232.28 | 3.91 |
| 37 | 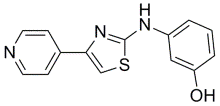 | 4 | 7.326 | 4 | Oc1cccc(Nc2nc(cs2)c3ccncc3)c1 | 269.32 | 3.24 |
| 38 | 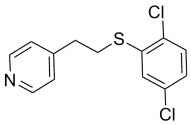 | 4 | 7.413 | 2 | Clc1ccc(Cl)c(SCCc2ccncc2)c1 | 284.23 | 4.65 |
| 39 | 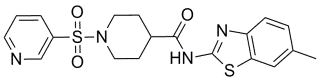 | 4 | 7.640 | 1 | Cc1ccc2nc(NC(=O)C3CCN(CC3)S(=O)(=O)c4cccnc4)sc2c1 | 416.52 | 2.74 |
| 40 | 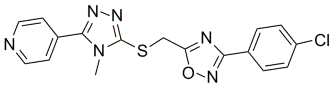 | 5 | 7.674 | 1 | Cn1c(SCc2onc(n2)c3ccc(Cl)cc3)nnc1c4ccncc4 | 384.85 | 4.58 |
| 41 | 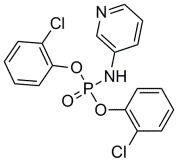 | 5 | 7.731 | 1 | Clc1ccccc1OP(=O)(Nc2cccnc2)Oc3ccccc3Cl | 395.20 | 4.16 |
| 42 | 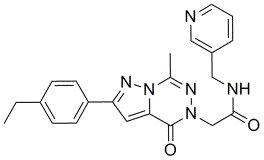 | 2 | 7.752 | 3 | CCc1ccc(cc1)c2cc3C(=O)N(CC(=O)NCc4cccnc4)N=C(C)n3n2 | 402.45 | 3.09 |
| 43 | 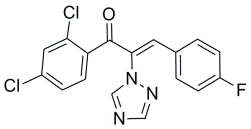 | 4 | 7.804 | 3 | Fc1ccc(\C=C(\C(=O)c2ccc(Cl)cc2Cl)/n3cncn3)cc1 | 362.21 | 4.27 |
| 44 | 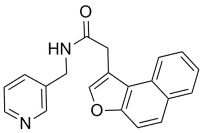 | 4 | 7.928 | 2 | O=C(Cc1coc2ccc3ccccc3c12)NCc4cccnc4 | 316.35 | 4.12 |
| 45 | 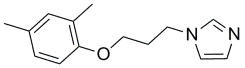 | 5 | 8.013 | 3 | Cc1ccc(OCCCn2ccnc2)c(C)c1 | 230.31 | 3.33 |
| 46 | 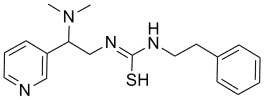 | 1 | 8.183 | 1 | CN(C)C(C\N=C(/S)\NCCc1ccccc1)c2cccnc2 | 328.48 | 3.47 |
| 47 | 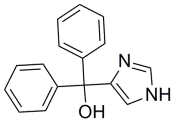 | 3 | 8.224 | 2 | OC(c1ccccc1)(c2ccccc2)c3c[nH]cn3 | 250.30 | 3.14 |
| 48 | 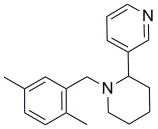 | 4 | 8.238 | 2 | Cc1ccc(C)c(CN2CCCCC2c3cccnc3)c1 | 280.41 | 4.31 |
| 49 | 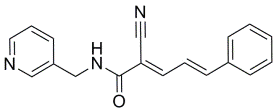 | 3 | 8.260 | 1 | O=C(NCc1cccnc1)\C(=C\C=C\c2ccccc2)\C#N | 289.33 | 2.81 |
| 50 | 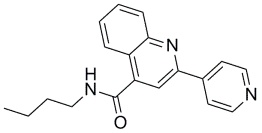 | 5 | 8.401 | 4 | CCCCNC(=O)c1cc(nc2ccccc12)c3ccncc3 | 305.37 | 3.96 |
| 51 | 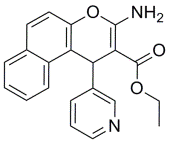 | 2 | 8.576 | 1 | CCOC(=O)C1=C(N)Oc2ccc3ccccc3c2C1c4cccnc4 | 346.38 | 3.93 |
| 52 | 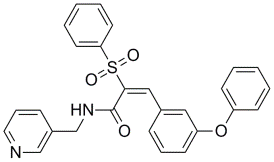 | 4 | 8.993 | 5 | O=C(NCc1cccnc1)\C(=C/c2cccc(Oc3ccccc3)c2)\S(=O)(=O)c4ccccc4 | 470.54 | 4.69 |
| 53 | 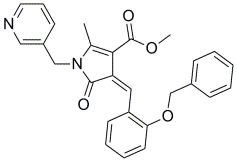 | 3 | 9.057 | 1 | COC(=O)C1=C(C)N(Cc2cccnc2)C(=O)/C/1=C\c3ccccc3OCc4ccccc4 | 440.49 | 5.00 |
| 54 | 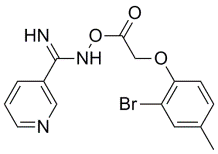 | 3 | 9.476 | 1 | Cc1ccc(OCC(=O)ONC(=N)c2cccnc2)c(Br)c1 | 364.19 | 2.27 |
| 55 | 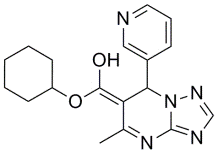 | 4 | 9.540 | 1 | CC1=Nc2ncnn2C(/C/1=C(\O)/OC3CCCCC3)c4cccnc4 | 339.39 | 2.31 |
| 56 | 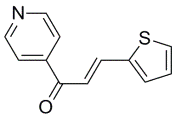 | 3 | 9.620 | 3 | O=C(\C=C\c1cccs1)c2ccncc2 | 215.27 | 2.49 |
| 57 | 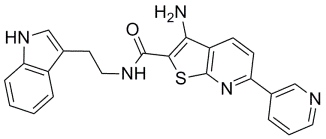 | 2 | 9.737 | 1 | Nc1c(sc2nc(ccc12)c3cccnc3)C(=O)NCCc4c[nH]c5ccccc45 | 413.50 | 4.79 |

^a^Toxicity was assessed as the highest percentage of host cell growth inhibition caused by compound treatment. Scores are assigned as **1**-<20%; **2**-21-30%; **3**- 31-40%; **4**-41-50% and 5->50% growth inhibition.

N/D - not determined
