# Supplementary material for: Diverse Inhibitor Chemotypes Targeting Trypanosoma cruzi CYP51
Source: PLoS Negl Trop Dis. 2012 Jul 31;6(7):e1736. doi: 10.1371/journal.pntd.0001736 (PMC3409115; doi:10.1371/journal.pntd.0001736)
Supplement: Table S5 — Second highest rated hit compound cluster. (DOCX) [file pntd.0001736.s006.docx]

**Table S5.** Second highest rated hit compound cluster

| Rank | Structure | HTS binding score | EC_50_, μM | K*_D_*, nM |
| --- | --- | --- | --- | --- |
| 2 | 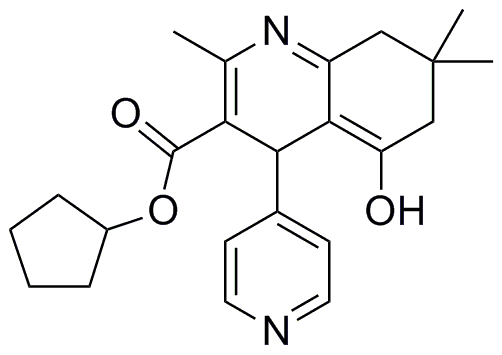 | 4 | 0.306 | N/D |
| 6 | 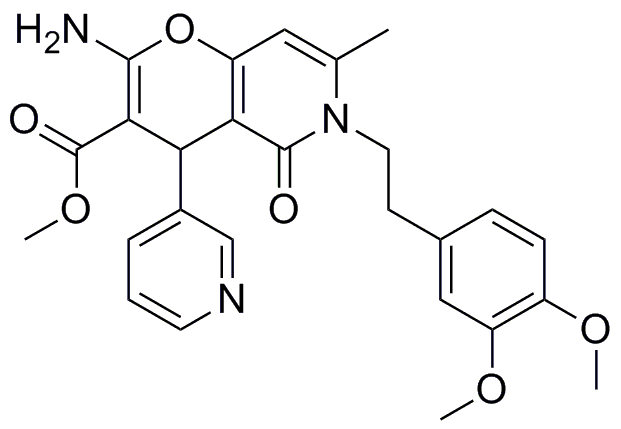 | 3 | 0.614^a^  (0.08±0.03)^b^ | 76±38 |
| 7 | 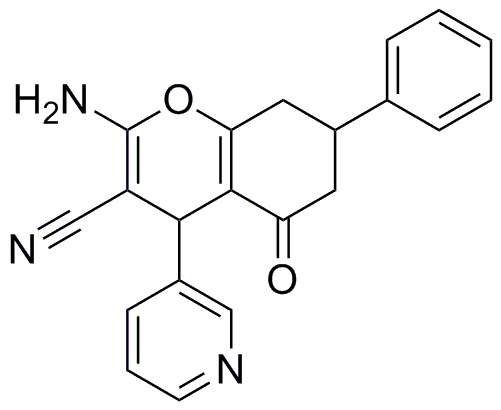 | 3 | 0.667 | N/D |
| 19 | 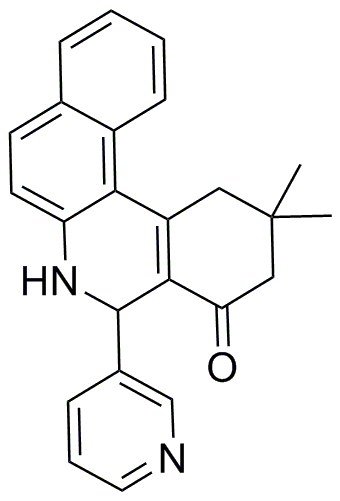 | 5 | 2.497 | ≤5 |
| 28 | 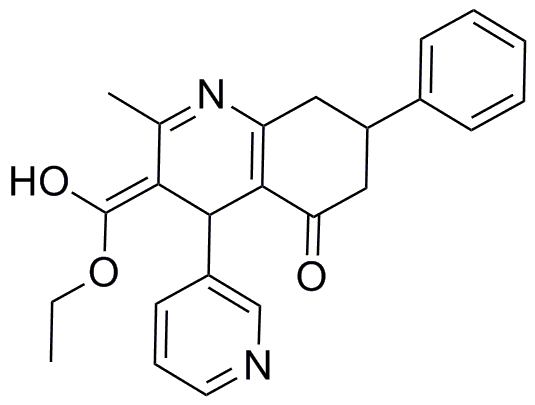 | 4 | 5.0 | N/D |
| 51 | 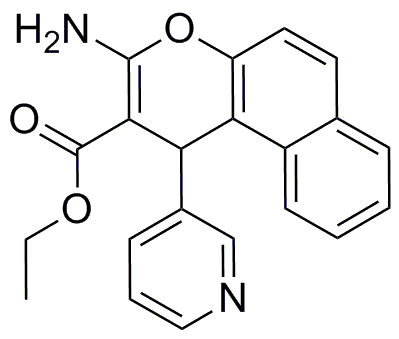 | 2 | 8.576 | N/D |
| Not ranked | 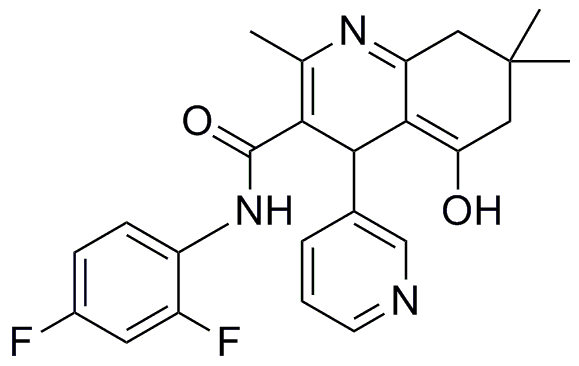 | 4 | None at ≤10 µM | N/D |
| Not ranked | 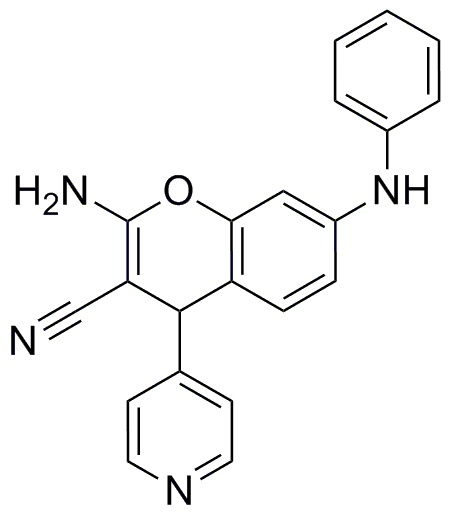 | 4 | None at ≤10 µM | N/D |
| Not ranked | 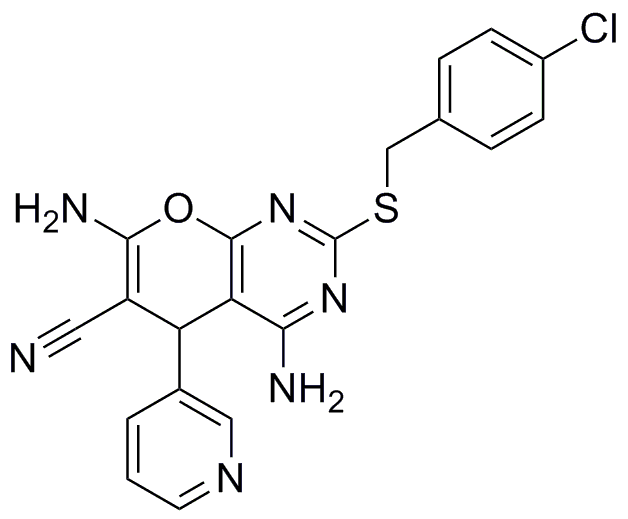 | 4 | None at ≤10 µM | N/D |
| Not ranked | 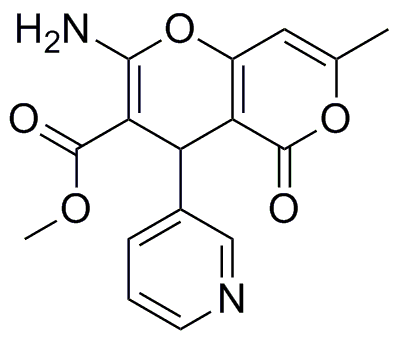 | 4 | None at ≤10 µM | N/D |
| Not ranked | 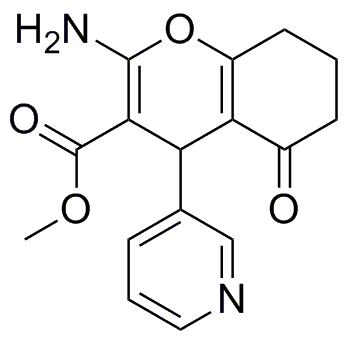 | 3 | None at ≤10 µM | N/D |
| Not ranked | 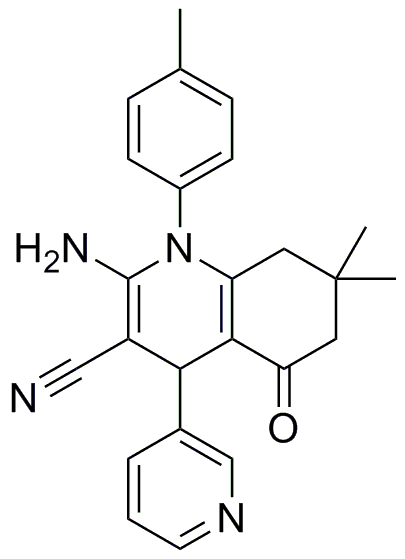 | 3 | None at ≤10 µM | N/D |
| Not ranked | 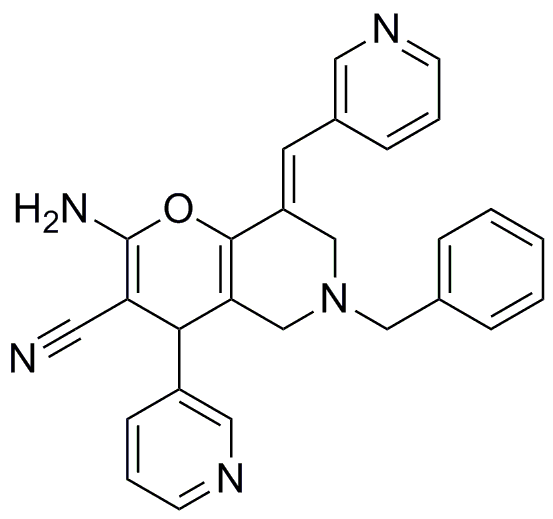 | 2 | None at ≤10 µM | N/D |
| Not ranked | 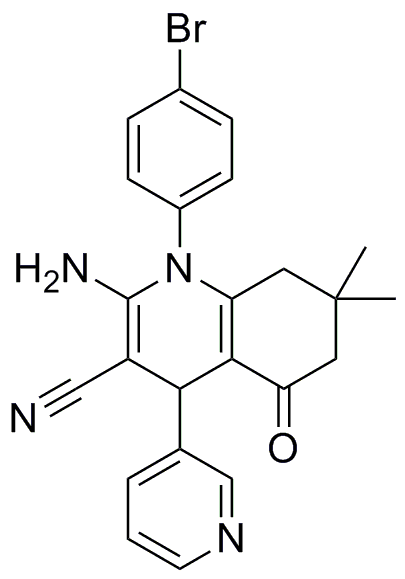 | 2 | None at ≤10 µM | N/D |
| Not ranked | 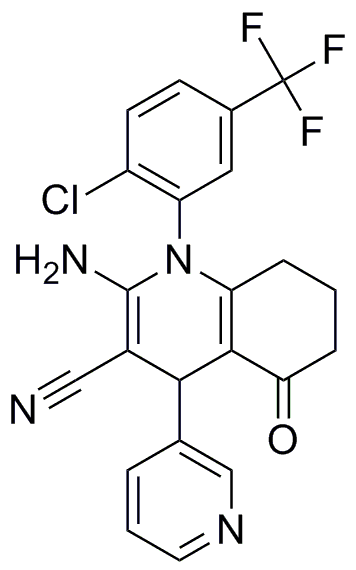 | 2 | None at ≤10 µM | N/D |
| Not ranked | 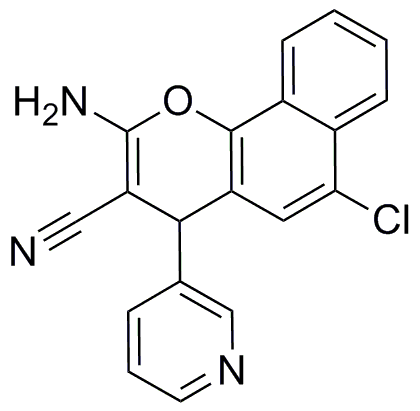 | 1 | None at ≤10 µM | N/D |
| Not ranked | 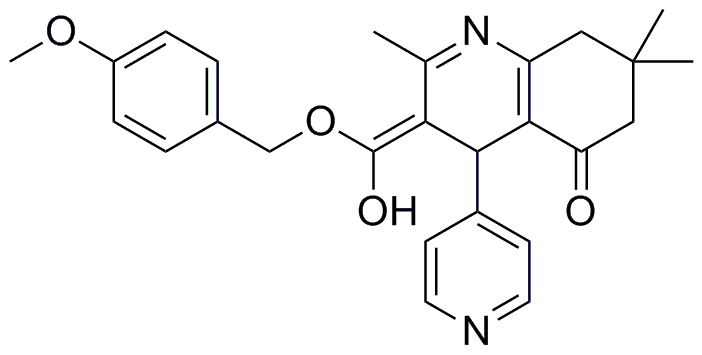 | 1 | None at ≤10 µM | N/D |
| Not ranked | 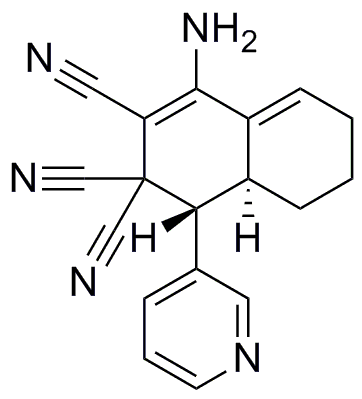 | 1 | None at ≤10 µM | N/D |
| Not ranked | 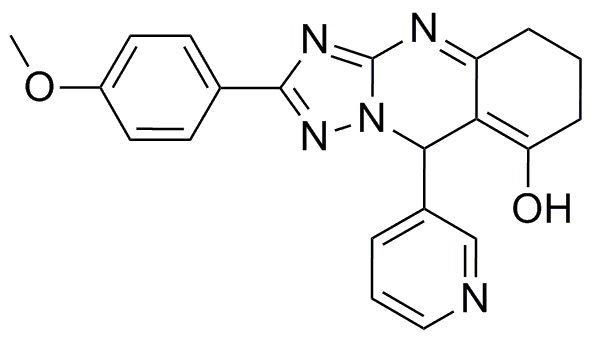 | 5 | None at ≤10 µM | N/D |
| Not ranked | 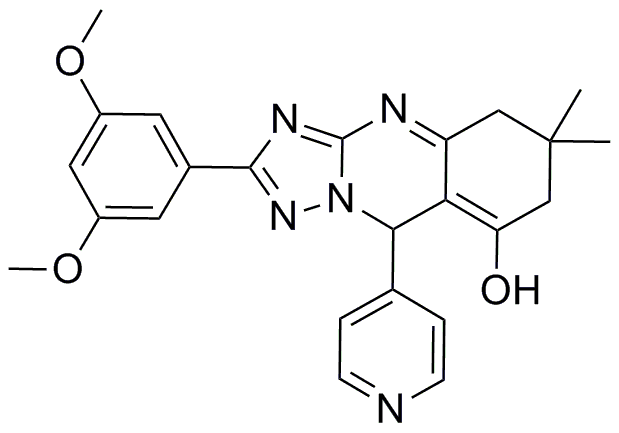 | 4 | None at ≤10 µM | N/D |
| Not ranked | 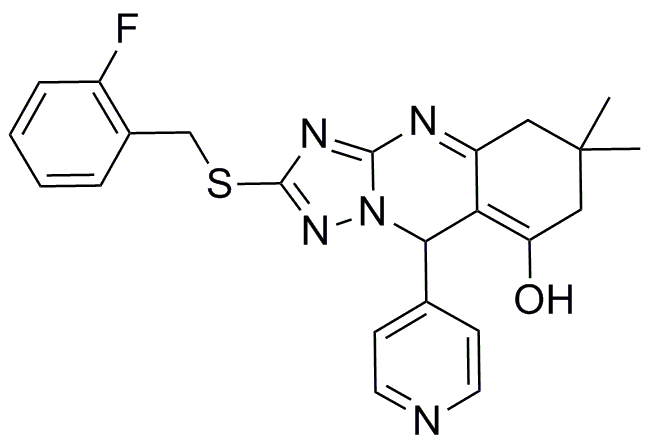 | 1 | None at ≤10 µM | N/D |

^a^EC_50_ values obtained against *T.cruzi* parasites in HTS assay used to rank order the hits

^b^In parenthesis are EC_50_ obtained in validation assays for individually purchased hits

N/A - not applicable

N/D - not determined
