# Supplementary material for: Diverse Inhibitor Chemotypes Targeting Trypanosoma cruzi CYP51
Source: PLoS Negl Trop Dis. 2012 Jul 31;6(7):e1736. doi: 10.1371/journal.pntd.0001736 (PMC3409115; doi:10.1371/journal.pntd.0001736)
Supplement: Table S6 — 32 individually validated hits. (DOCX) [file pntd.0001736.s007.docx]

**Table S6.** 32 individually validated hits

| ***T. cruzi*-active hits** | | | |
| --- | --- | --- | --- |
| **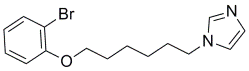** | **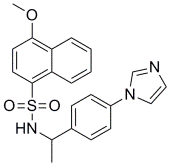** | **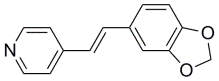** | 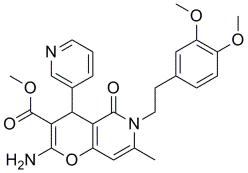 |
| **1**^a^ (2)^b^ 0.017 μM^c^ | **3** (4) 0.20 μM | **5** (4) 0.26 μM | **6** (3) 0.08 μM |
| 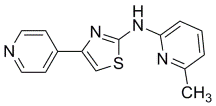 | 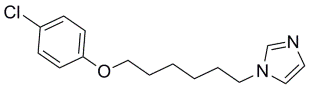 | 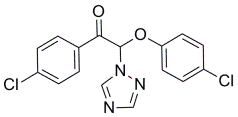 | 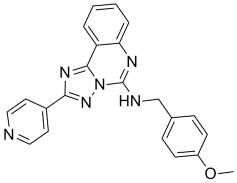 |
| **9** (4) 0.58 μM | **11** (5) 0.76 μM | **13** (5) 1.30 μM | **15** (5) 1.93 μM |
| **CYP51 binding hits** | | | |
| 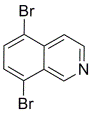 | 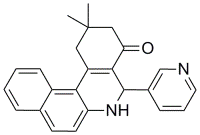 | 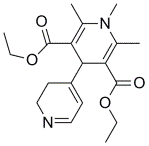 | 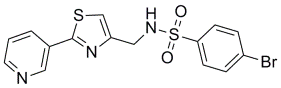 |
| (4) | (5) | (5) | (5) |
| 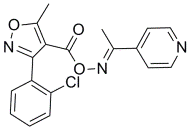 | 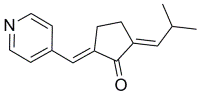 | 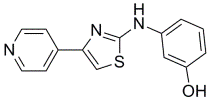 | 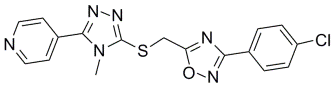 |
| (5) | (4) | (4) | (5) |
| 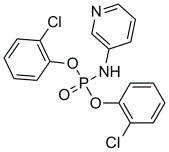 | 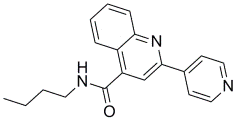 | 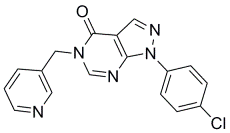 | 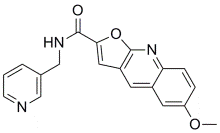 |
| (5) | (5) | (5) | (5) low solubility |
| 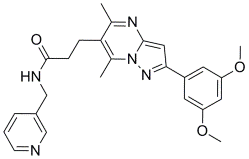 | 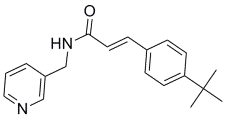 | 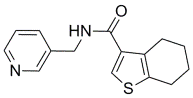 | 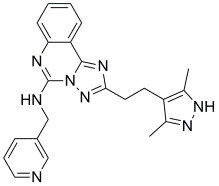 |
| (5) | (5) | (5) | (5) |
| 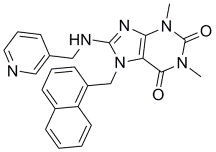 | 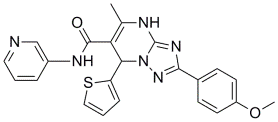 | 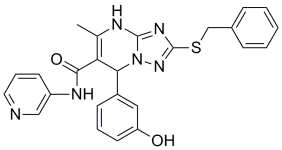 | 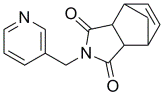 |
| (4) | (4) | (4) | (5) low solubility |
| 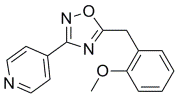 | 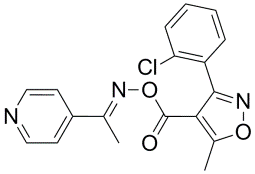 | 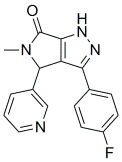 | 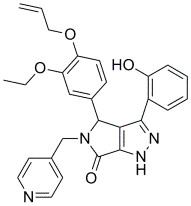 |
| (4) | (5) | (5) | (5) |

^a^ Rank in *T. cruzi* assay; ^b^HTS binding score is given in parentheses; ^c^EC_50_ versus *T. cruzi* parasites are in μM.
