# Supplementary material for: Diverse Inhibitor Chemotypes Targeting Trypanosoma cruzi CYP51
Source: PLoS Negl Trop Dis. 2012 Jul 31;6(7):e1736. doi: 10.1371/journal.pntd.0001736 (PMC3409115; doi:10.1371/journal.pntd.0001736)
Supplement: Alternative Language Abstract S1 — Manuscript Abstract in Portuguese. (DOC) [file pntd.0001736.s008.doc]

**Introdução.** A doença de Chagas, considerada pela OMS e pelo NIH como uma doença tropical negligenciada (DTN), é endêmica na América Latina e infecção emergente nos EUA e Espanha, como resultado dos movimentos demográficos. Embora seja uma das principais causas de morbidade e mortalidade por insuficiência cardíaca e pesado fardo econômico nas regiões afetadas, a doença de Chagas atrai pouca atenção da indústria farmacêutica por causa de escassos incentivos econômicos. Assim, a descoberta e o desenvolvimento de novas rotas para a quimioterapia para a doença de Chagas torna-se uma prioridade clara.

**Metodologia/ Principais achados.** A similaridade entre os requisitos de esteróis de membrana entre fungos patogênicos e o parasita *Trypanosoma cruzi*, agente causador da cardiomiopatia chagásica humana, levou ao reaproveitamento de azoles anti-fúngicos e inibidores triazólicos da esterol 14 α-demetilase (CYP51) para o tratamento da Doença de Chagas. Para diversificar a seleção terapêutica dos candidatos a drogas anti-Chagásicas, exploramos uma abordagem complementar que incluiu o teste direto do sítio ativo da CYP51 de *T. cruzi* contra uma biblioteca de pequenas moléculas sintéticas. A triagem em formato de alta vazão baseada em alvos moleculares reduziu uma biblioteca de aproximadamente 104.000 pequenas moléculas para 185 compostos ativos (“hits”) para ligação com a enzima com valores de K*_D_* estimados na faixa nanomolar, enquanto a validação cruzada em mioblastos esqueléticos infectados pelo *T. cruzi* resultou em 57 “hits” ativos com EC_50_ <10 μM. Dois conjuntos de “hits” se superpuseram parcialmente. O melhor composto inibiu a infecção pelo *T. cruzi* com EC_50_ de 17 nM e foi tripanocida a 40 nM.

**Conclusões/Significância.** Os “hits” identificados neste trabalho são estruturalmente diversos, demonstrando que CYP51 é um alvo bastante permissivo a pequenas moléculas. A análise quimioinformática dos “hits”sugeriu que a farmacologia da CYP51 é similar a outros alvos terapêuticos da família citocromo P450, como por exemplo, tromboxano sintase (CYP5), ω-hidroxilases de ácidos graxos (CYP4), 17-α-hidroxilase/17,20-liase (CYP17) e aromatase (CYP19). Surpreendentemente, alta similaridade é sugerida a glutaminil-peptideo ciclotransferase, que não é relacionada a CYP51 por sequência nem por estrutura. Compostos líderes desenvolvidos por companhias farmacêuticas contra esses alvos também podem ser explorados para eficácia contra *T. cruzi*.
